# Mortality and Clinical Interventions in Critically ill Patient With Coronavirus Disease 2019: A Systematic Review and Meta-Analysis

**DOI:** 10.3389/fmed.2021.635560

**Published:** 2021-07-23

**Authors:** Zhicheng Qian, Shuya Lu, Xufei Luo, Yaolong Chen, Ling Liu

**Affiliations:** ^1^Jiangsu Provincial Key Laboratory of Critical Care Medicine, Department of Critical Care Medicine, Zhongda Hospital, School of Medicine, Southeast University, Nanjing, China; ^2^Department of Critical Care Medicine, Affiliated Hospital of North Sichuan Medical College, Nanchong, China; ^3^Evidence-Based Medicine Center, School of Basic Medical Sciences, Lanzhou University, Lanzhou, China; ^4^Department of Pediatric, Sichuan Provincial People's Hospital, University of Electronic Science and Technology of China, Chengdu, China; ^5^School of Public Health, Lanzhou University, Lanzhou, China; ^6^Institute of Health Data Science, Lanzhou University, Lanzhou, China; ^7^World Health Organization Collaborating Centre for Guideline Implementation and Knowledge Translation, Lanzhou, China; ^8^Key Laboratory of Evidence Based Medicine and Knowledge Translation of Gansu Province, Lanzhou University, Lanzhou, China

**Keywords:** mortality, critically ill patients, COVID-19, clinical interventions, supportive care

## Abstract

**Objective:** The aims of this systematic review and meta-analysis were to summarize the current existing evidence on the outcome of critically ill patients with COVID-19 as well as to evaluate the effectiveness of clinical interventions.

**Data Sources:** We searched MEDLINE, the Cochrane library, Web of Science, the China Biology Medicine disc, China National Knowledge Infrastructure, and Wanfang Data from their inception to May 15, 2021. The search strings consisted of various search terms related to the concepts of mortality of critically ill patients and clinical interventions.

**Study Selection:** After eliminating duplicates, two reviewers independently screened all titles and abstracts first, and then the full texts of potentially relevant articles were reviewed to identify cohort studies and case series that focus on the mortality of critically ill patients and clinical interventions.

**Main Outcomes and Measures:** The primary outcome was the mortality of critically ill patients with COVID-19. The secondary outcomes included all sorts of supportive care.

**Results:** There were 27 cohort studies and six case series involving 42,219 participants that met our inclusion criteria. All-cause mortality in the intensive care unit (ICU) was 35% and mortality in hospital was 32% in critically ill patients with COVID-19 for the year 2020, with very high between-study heterogeneity (*I*^2^ = 97%; *p* < 0.01). In a subgroup analysis, the mortality during ICU hospitalization in China was 39%, in Asia—except for China—it was 48%, in Europe it was 34%, in America it was 15%, and in the Middle East it was 39%. Non-surviving patients who had an older age [−8.10, 95% CI (−9.31 to −6.90)], a higher APACHE II score [−4.90, 95% CI (−6.54 to −3.27)], a higher SOFA score [−2.27, 95% CI (−2.95 to −1.59)], and a lower PaO_2_/FiO_2_ ratio [34.77, 95% CI (14.68 to 54.85)] than those who survived. Among clinical interventions, invasive mechanical ventilation [risk ratio (RR) 0.49, 95% CI (0.39–0.61)], kidney replacement therapy [RR 0.34, 95% CI (0.26–0.43)], and vasopressor [RR 0.54, 95% CI (0.34–0.88)] were used more in surviving patients.

**Conclusions:** Mortality was high in critically ill patients with COVID-19 based on low-quality evidence and regional difference that existed. The early identification of critical characteristics and the use of support care help to indicate the outcome of critically ill patients.

## Introduction

With the rapid spread of coronavirus disease 2019 (COVID-19) globally, as of June 2, 2021, a total of 171,222,477 confirmed cases had been reported in 215 countries, areas, or territories, and COVID-19 has been responsiblefor at least 3,686,142 deaths (1). Critically ill patients are always companied by a high risk of lives, which may be complicated by an uncontrolled systemic inflammatory response leading to acute respiratory distress syndrome (ARDS) and multiple organ dysfunction. Patients with ARDS and requirement for respiratory support need urgently to be transferred to the intensive care unit (ICU). It is reported that nasal cannula or mask, high-flow nasal cannula, non-invasive ventilation (NIV), invasive mechanical ventilation (IMV), and veno-venous extracorporeal membrane oxygenation (VV-ECMO) were widely used in COVID-19 according to the severity of respiratory dysfunction (2–4). Cardiac injury is common in COVID-19, with an incidence of 36% and closely related to a higher risk of mortality (5). It is reported that, in a systematic review and meta-analysis, the pooled incidence of acute kidney injury (AKI) was 28.6% among hospitalized COVID-19 patients from the USA and Europe and 5.5% among patients from China. Kidney replacement therapy (KRT) was used in 20.6% of patients admitted to the intensive care unit (6).

As is universally known, the mortality of critically ill patients is higher than that of ordinary patients. A systematic review reported that the summary estimate for all-cause mortality was 10% for adult patients with COVID-19 and 34% for critically ill patients within minor countries (7). In order to gain a clearer picture of the mortality of critically ill patients within major countries and clinical interventions or supportive care for organ dysfunction in the ICU, we meta-analyzed the relevant literature. The results may provide a narrative for the mortality of critically ill patients with COVID-19 as well as the effect of clinical characteristics and interventions between surviving and non-surviving patient groups.

## Methods

This systematic review was performed in compliance with the Centre of Reviews and Dissemination guidelines (8) and reported according to the Preferred Reporting Items for Systematic Reviews and Meta-analysis (PRISMA) statement (9). In order to complete the systematic review and provide some references for clinical intervention during COVID-19 as soon as possible, this review was not registered.

### Eligibility Criteria

We included studies that focused on the mortality of critically ill patients with laboratory-confirmed COVID-19, clinical characteristics, and interventions or supportive care of organ dysfunction.

We included original studies that fulfill the following criteria: (1) the type of study was cohort, case–control, or case–series designs, (2) the study topic was related to the mortality, clinical characteristics, and interventions or supportive care of critically ill patients with COVID-19, which is defined as a positive result of a real-time reverse transcriptase–polymerase chain reaction (RT-PCR) assay of nasal and pharyngeal swabs (10), and (3) the study was published or posted in English or Chinese. We excluded duplicates, conference abstracts, letters, and studies for which we could not access the full text and missing data of outcomes. In order to avoid a small size, only studies of more than 50 patients were included. If there were two or more studies that included the same population, only the study with the largest sample size was chosen.

In this review, the primary outcome was the mortality of critically ill patients with COVID-19. The secondary outcomes included all sorts of supportive care, including non-invasive respiratory support, IMV, KRT, and vasopressor. Critically or severely ill patients were defined as those patients who were admitted to the ICU or required respiratory support. Surviving patients were defined as those discharged from the ICU or hospital or who remained hospitalized. Non-surviving patients were defined as those who died in the ICU or hospital. Immunoregulation therapy includes corticosteroids, interferon, and intravenous immunoglobulin G.

### Search of Studies

Two reviewers (ZQ and SL) carried out the search independently in the following six electronic databases from their inception to May 15, 2021: MEDLINE (*via* PubMed), the Cochrane library, Web of Science, China Biology Medicine disc, China National Knowledge Infrastructure, and Wanfang Data. The main terms were “mortality,” “critically ill patient,” “severely ill patient,” “novel coronavirus,” “2019-novel coronavirus,” “Novel CoV,” “SARS-CoV-2,” “COVID-19,” “2019-CoV,” “invasive mechanical ventilation,” “high flow nasal cannula,” “non-invasive ventilation,” “extracorporeal membrane oxygenation,” “renal replacement therapy,” “kidney replacement therapy,” “vasopressor,” and so on (the details of the search strategy can be found in Supplementary File 1). Moreover, we also searched the clinical trial registry platforms, the Google Scholar, the reference lists of the identified reviews, and the preprint platforms [including SSRN (https://www.ssrn.com/index.cfm/en/), medRxiv (https://www.medrxiv.org/), and bioRxiv (https://www.biorxiv.org/)] for further potential studies.

### Selection of Studies

After eliminating duplicates by using EndNote X9.3.2 software, two reviewers independently screened all titles and abstracts first, and then the full texts of potentially relevant articles were reviewed to identify the final inclusion. Discrepancies were settled by discussion or consultation with a third reviewer. All reasons for exclusion of ineligible studies were recorded, and the process of study selection was documented using a PRISMA flow diagram (11).

### Data Extraction

Two reviewers (ZQ and SL) extracted data independently with a standard data collection form. Any disagreements were resolved by consensus, and a third reviewer (XL) checked the consistency and accuracy of all data. The following data and information were extracted for each included study: basic information (title, first author, publication year, funding, and study design), information on the participants (sample size, age, and inclusion/exclusion criteria of participants), details of the intervention and control conditions, outcome information [for dichotomous data, we abstracted the number of events and total participants per group; for continuous data, we abstracted the means, standard deviations (SD), and number of total participants per group].

### Risk of Bias in Individual Studies

Two reviewers (ZQ and SL) assessed the potential risk of bias of each included study independently. Discrepancies were resolved by discussion and consensus with a third researcher (XL). We assessed the risk of bias in cohort studies using Newcastle–Ottawa Scale (12), which contains eight domains: representativeness of exposure cohorts, selection of non-exposure cohorts, determination of exposure, outcome events that did not occur before study initiation, comparability of cohort based on design or analysis, assessment of outcome events, adequacy of follow-up time, and completeness of follow-up. For case series, we used the Joanna Briggs Institute critical appraisal checklist for case series (13), which consists of 10 domains. Each domain was graded as one sore if reported.

### Statistical Analysis

All statistical analyses were performed using RStudio, version 1.3.1056. Comparable data from studies with one outcome were pooled using forest plots according to the Cochrane Handbook by using random-effects model separately (14). Mortality in the ICU and in hospital was used for a detailed description. A subgroup analysis was performed according to different regions. For dichotomous outcomes, we calculated the risk ratios (RR) and the corresponding 95% confidence intervals (CI) and *P*-values. For continuous outcomes, we calculated the standardized mean difference and its corresponding 95% CI if means and SD were reported. Furthermore, 95% prediction interval (PI) was used to evaluate the range that, we assert with 95% certainty, will fall into during a future validation test. We reported the effect size with 95% CI by using random-effects models. Two-sided *P* < 0.05 were considered statistically significant. Heterogeneity was defined as *P* < 0.10 and *I*^2^ >50%.When effect sizes could not be pooled due to only one study for a comparison, we reported the study findings narratively. We used sensitivity analyses to evaluate the stability of mortality outcomes of the included studies. For a result that included more than 10 studies, publication bias was tested by visual funnel plots.

### Quality of the Evidence

The quality of evidence for each outcome was assessed by using the Grading of Recommendations Assessment, Development and Evaluation (GRADE) approach. The judgments of quality for specific outcomes were based on five main factors: study design and execution limitations, inconsistency, indirectness, imprecision of results (random-effects model), and publication bias across all studies (15, 16). The quality of evidence for each outcome was graded as high, moderate, low, or very low (17) and presented in “GRADE Evidence Profiles” (18).

## Results

### Search Results

**Figure 1:**
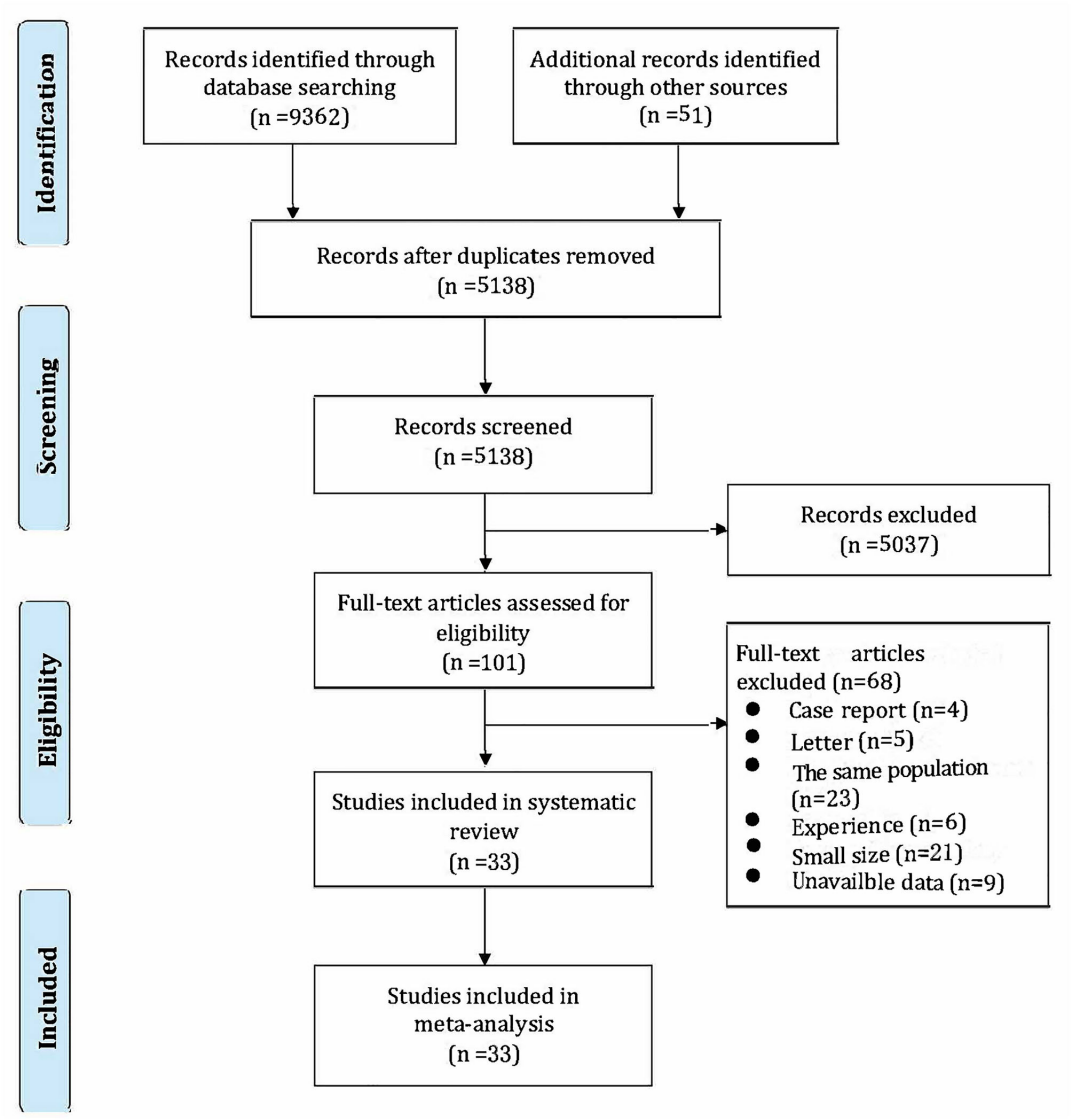
Study selection process and results.

The literature search retrieved 9,362 records through database searching and 51 additional records through other sources, which included 36 from the Google Scholar and 15 from preprint platforms. After removing duplicates, we screened the titles and abstracts of 5,138 records and reviewed the full text of 101 articles. Finally, we included 33 studies (cohort studies and case–series) (19–51) that reported either the mortality of critically ill patients or the clinical interventions between surviving and non-surviving patients with COVID-19 (Figure 1). All of them were published in English.

### The Characteristics of the Included Studies

The basic characteristics of the included studies of the mortality of critically ill patients are summarized in Table 1 (Supplementary File 2). These 28 studies involving 40,195 participants were admitted between January 1 and December 30, 2020, which covered Asia, Europe, and America. Of the 28 studies, 19 were single-center studies and nine were multi-center studies in design. Mortality was demonstrated and concluded with a follow-up of more than 7 days and expressed as mortality in the ICU or in hospital. Among 33 studies, 17 studies (22, 25, 50, 51) with 6,414 participants compared clinical interventions between surviving and non-surviving patients. All studies assessed the risk of bias with scores of 3–9, indicating low to high quality (Supplementary File 3). A visual analysis of the funnel plot indicated that no publication bias was suspected in the results of age and mortality in the ICU. The results of IMV, PaO_2_/FiO_2_ ratio, and SOFA source were suggestive of publication bias (Supplementary File 4).

**Table 1:** Basic characteristics of included studies of mortality of critically ill patients.

**Region**	**Nation**	**Study**	**No. patients**	**Study design**	**Single- or multi-center**	**Date**	**Follow-up**	**Outcome**
Asia	China	Xie et al. (51)	733	Retrospective case series	Multi-center	Jan. 1 to Feb. 29	28-days	394 patients died
	China	Li et al. (38)	268	Retrospective, cohort study	Single-center	Jan. 26 to Feb. 5	32-days	87 patients died, 85 discharged from hospital
	China	Hu et al. (33)	55	Retrospective case series	Single-center	Jan. 8 to Mar. 12	28-days	16 patients died, 33 discharged home. Six transferred to isolation wards
	China	Chen et al. (24)	192	Retrospective case series	Single-center	Jan. 28 to Mar. 13	Until Mar. 13	50 died in the hospital and 142 were discharged
	China	Geng et al. (29)	123	Retrospective observational study	Single-center	Feb. 9 to Apr. 6	Until Apr. 6	57 died in ICU hospitalization and 66 were discharged
	Pakistan	Rahim et al. (44)	204	Cross-sectional study	Single-center	Apr. 1 to Aug. 31	Until Aug. 31	157 died in ICU hospitalization and 47 shifting from the ICU to a general isolation ward
	India	Mahendra et al. (39)	560	Retrospective observational study	Single-center	Jun. 1 to Oct. 30	30-days	306 died in hospital
	Thailand	Sivakorn et al. (52)	60	Prospective observational study	Single-center	Jan. 1 to 31	Until Jan. 31	12 died and 48 patients were alive at ICU discharge.
Middle East	Qatar	Najim et al. (41)	60	Prospective observational study	Single-center	Jun. 26 to Aug. 5	60-days or died or discharged from the ICU	Seven died
	Iran	Vahedi et al. (48)	133	Retrospective cohort study	Single-center	Feb. 1 to Jun. 30	Until Jun. 30	77 died in ICUs and other patients were recovered or discharged from ICUs.
	Saudi Arabia	Alharthy et al. (20)	352	Retrospective observational study	Single-center	Mar. 20 to May 31	28-days	113 died in ICU hospitalization
	Libya	Elhadi et al. (26)	465	Prospective cohort study	Multi-center	May 29 to Dec. 30	60-days	281 died in ICU and 184 discharged alive
Europe	Europe	Wendel Garcia et al. (50)	398	Prospective cohort study	Multi-center	Mar. 13 to Apr. 22	40-days	97 patients died and 301 discharged
	France	Fond et al. (27)	14,351	Retrospective cohort study	Multi-center	Feb. 1 to Jun. 9	until Jun. 9	3,790 died in hospital
	Spain	Rodríguez et al. (45)	2,022	Prospective observational	Multi-center	Feb. 22 to May 11	90-days	660 died in ICU and 1,362 discharged from ICU
	Italy	Gamberini et al. (28)	391	Retrospective observational study	Multi-center	Feb. 22 to May 4	Until May 15	141 died in ICU hospitalization, 39 still in ICU
	Italy	Carpagnano et al. (23)	78	Retrospective observational study	Single-center	Mar. 11.to Apr. 27	Until Apr. 27	35 patients died during hospitalization, 43 discharge from the ICU
	Italy	Grasselli et al. (30)	1,581	Retrospective case series	Multi-center	Feb. 20 to Mar. 18	7-days	405 patients died, 920 still in the ICU, 256 discharged
	Sweden	Jonmarker et al. (35)	152	Retrospective observational study	Single-center	Mar. 1 to Apr. 31	28-days	43 died in ICU
	Sweden	Järhult et al. (34)	92	Prospective observational	Single-center	Mar. 1 to Jun. 30	30-days	21 died in ICU
	Netherland	Aleva et al. (19)	50	Retrospective case series	Single-center	Mar. 9 to Apr. 7	86-days	13 patients died, 37 survived and discharged from ICU
	Netherland	Vogels et al. (49)	114	Retrospective observational study	Single-center	Mar. 1 to Jun. 4	28-days	31 died in ICU hospitalization
	Turkey	Gunduz et al. (31)	209	Retrospective observational study	Single-center	Mar. 24 to Jul. 6	Until their outcomes	82 died in ICU hospitalization
	Serbia	Popadic et al. (43)	160	Retrospective observational study	Single-center	Jun. 23 to Oct. 2	Until their outcomes	96 died in ICU hospitalization, 64 lived
	Greece	Routsi et al. (47)	50	Prospective observational study	Single-center	Mar. 11 to Apr. 27	Until Apr. 27	16 patients died, one still in the ICU, 33 discharged
America	US	Gupta et al. (32)	3,924	Retrospective cohort study	Multi-center	Mar. 4 to May 10	30-days	1,544 patients died, 2,058 discharged alive, 322 remained hospitalized.
	Canada	Mitra et al. (40)	117	Retrospective case series	Multi-center	Feb. 21 to Apr. 14	21-days	18 patients died, 12 remained in ICU, 16 discharged from ICU but remained in hospital, and 71 discharged home.
	Brazil	Kurtz et al. (36)	13,301	Retrospective cohort study	Multi-center	Feb. 27 to Oct. 28	60-days	1,785 patients died during hospitalization, 82 remained hospitalized

### Clinical Outcome of Critically Ill Patients

**Figure 2:**
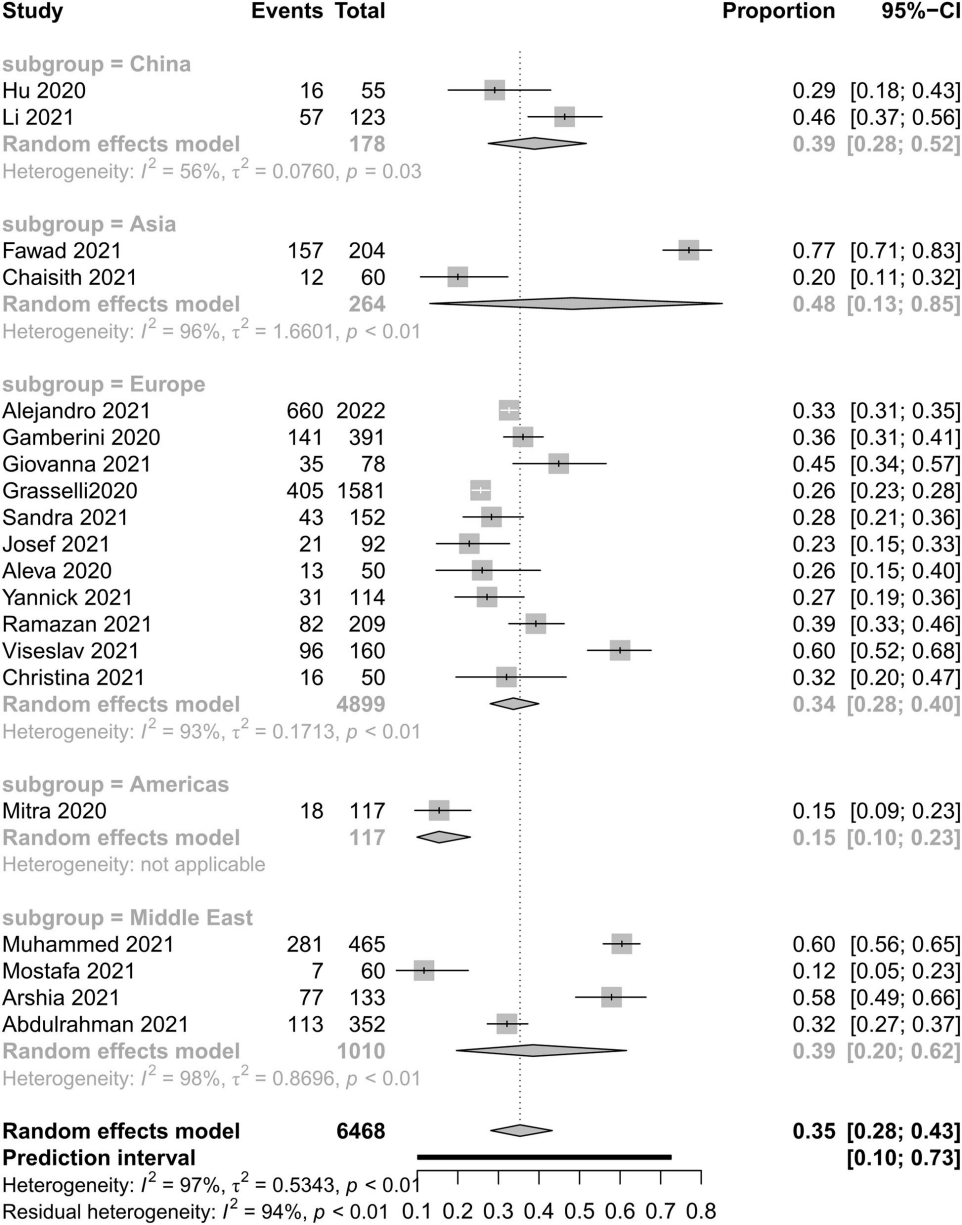
All-cause mortality in intensive care unit with COVID-19.

**Figure 3:**
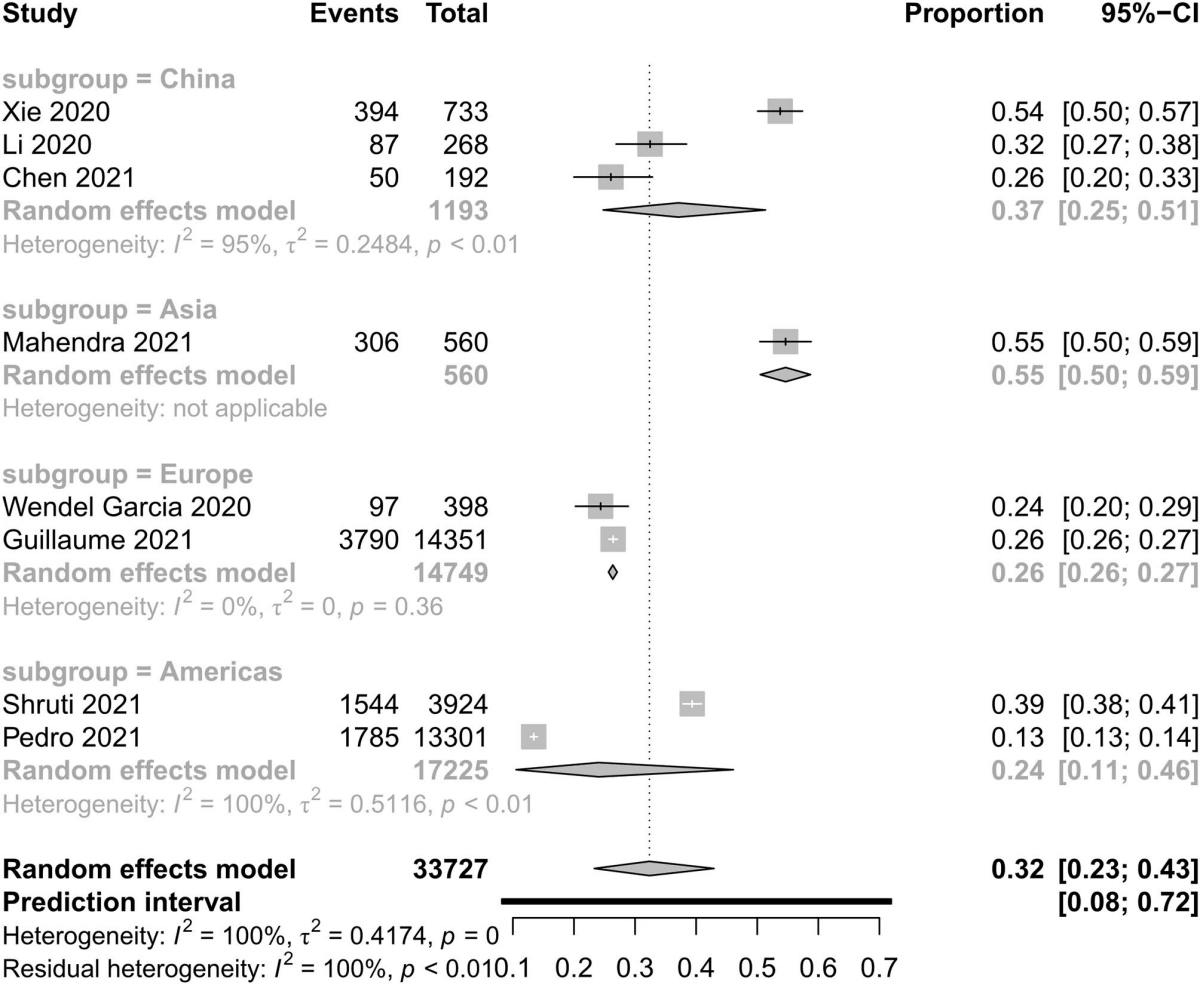
All-cause mortality in hospital with COVID-19.

Figures 2, 3 show all-cause mortality in the ICU and in hospital as per peer-reviewed studies from countries around the world. In the present study, all-cause mortality in the ICU was 35% in critically ill patients (95% PI, 10–73%) with very high between-study heterogeneity. In a subgroup analysis, the mortality in China was 39%, in Asia—except for China—it was 48%, in Europe it was 34%, in America it was 15%, and in the Middle East it was 39%. For mortality in hospital, all-cause mortality was 32% (95% PI, 8–72%) with very high between-study heterogeneity. In a subgroup analysis, the mortality in China was 37%, in Asia—except for China—it was 55%, in Europe it was 26%, and in America it was 24%.

### Basic Clinical Characteristics Between Two Different Outcome Groups

**Figure 4:**
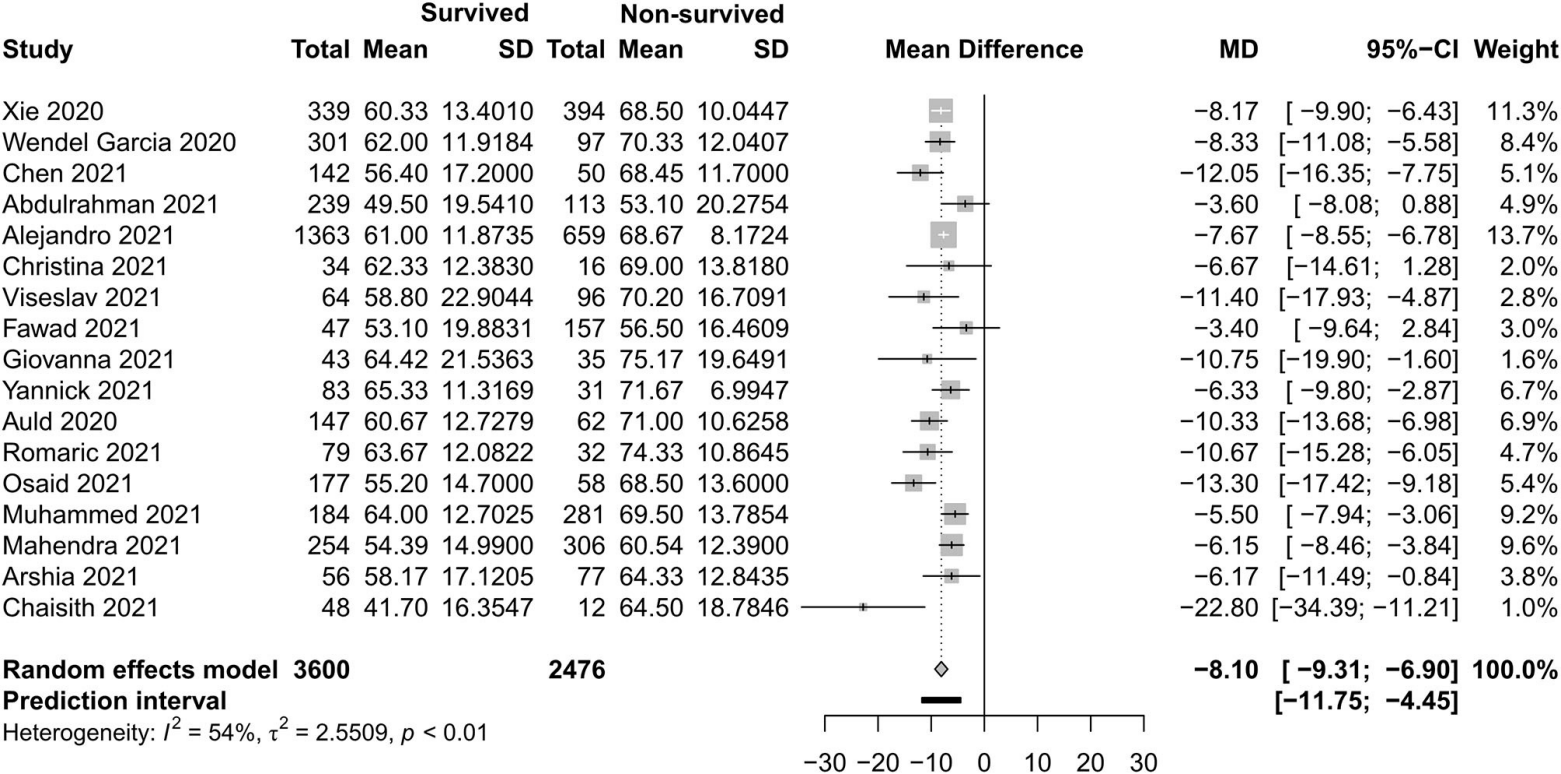
Mean difference of age between survived and non-survived patients.

**Figure 5:**
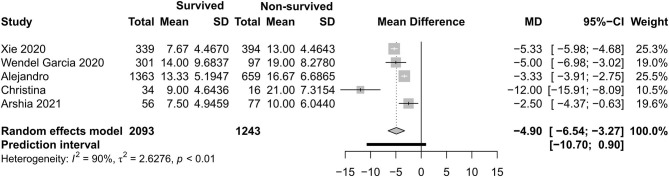
Mean difference of Acute Physiological and Chronic Health Evaluation II score between survived and non-survived patients.

**Figure 6:**
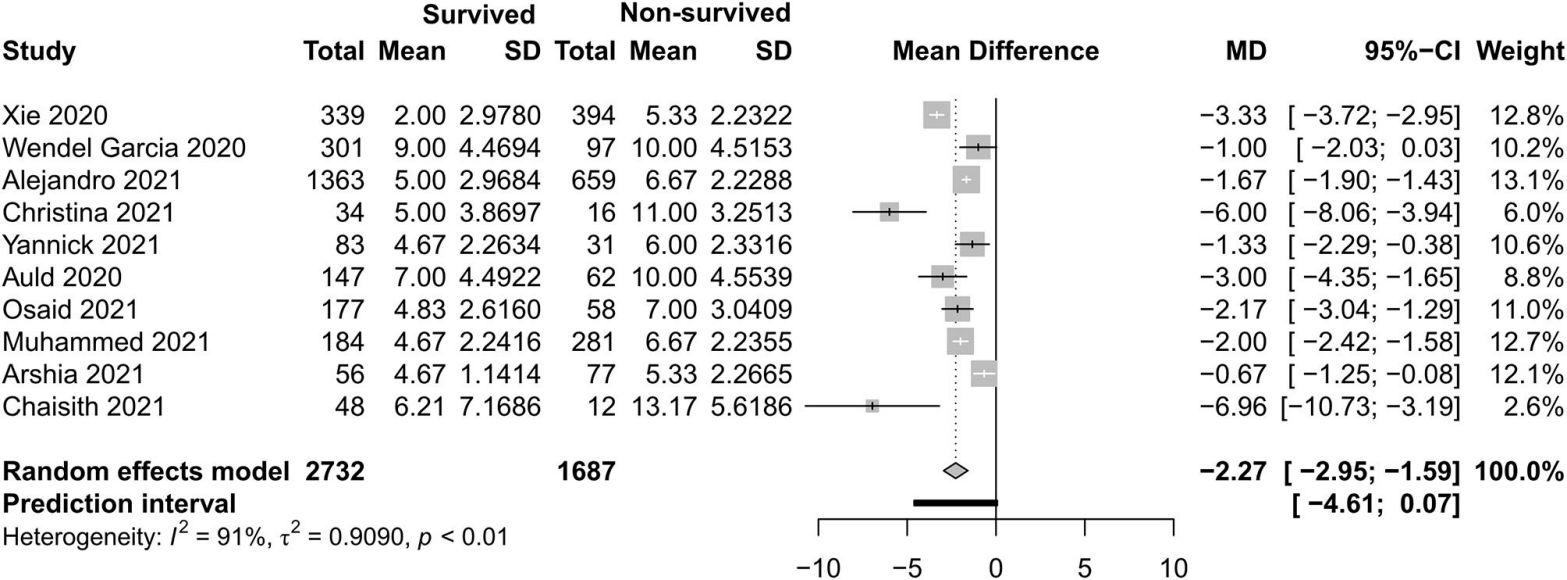
Mean difference of Sequential Organ Failure Assessment score between survived and non-survived patients.

**Figure 7:**
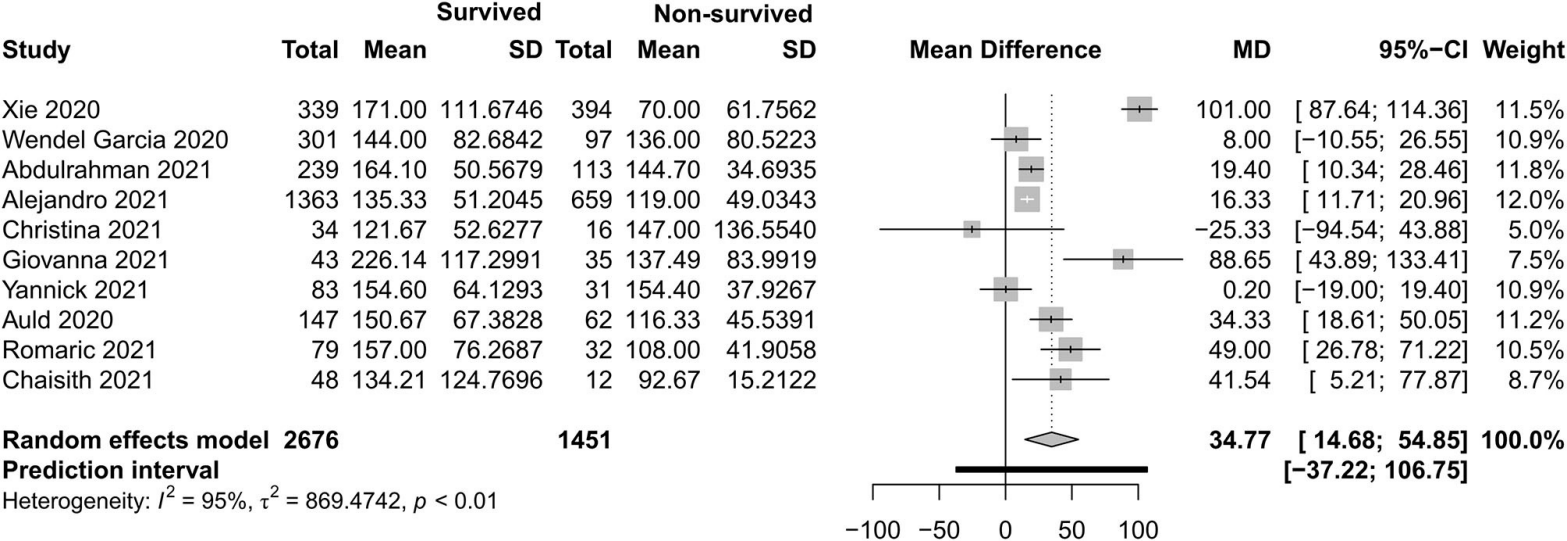
Mean difference of the PaO_2_/FiO_2_ ratio between survived and non-survived patients.

Figures 4–7 show the basic clinical characteristics including age, acute physiological and chronic health evaluation II (APACHE II) score, sequential organ failure assessment (SOFA) score, and PaO_2_/FiO_2_ ratio between surviving and non-surviving patients. Patients who did not survive had an older age [−8.10, 95% CI (−9.31 to −6.90)], a higher APACHE II score [−4.90, 95% CI (−6.54 to −3.27)], a higher SOFA score [−2.27, 95% CI (−2.95 to −1.59)], and a lower PaO_2_/FiO_2_ ratio [34.77, 95% CI (14.68 to 54.85)] than those who survived.

### Respiratory Support Care

**Figure 8:**
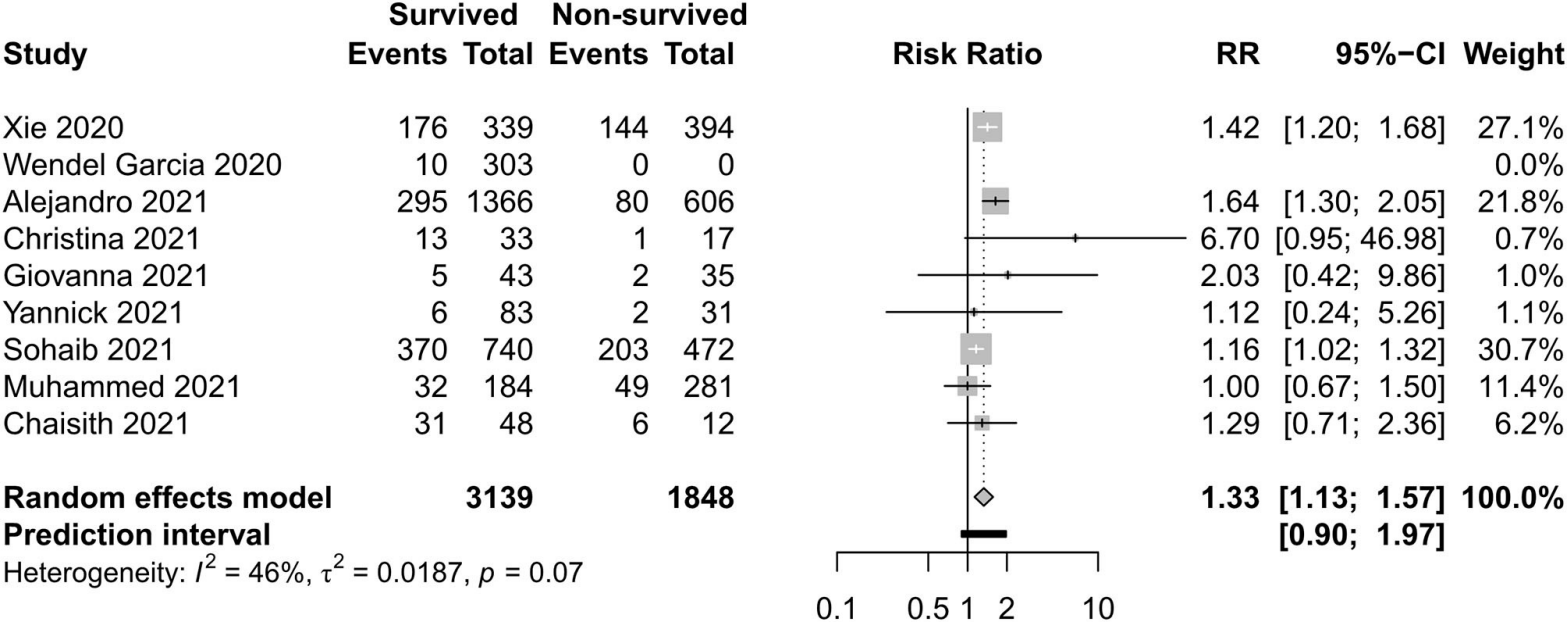
Risk ratio of high-flow nasal oxygenation between survived and non-survived patients.

**Figure 9:**
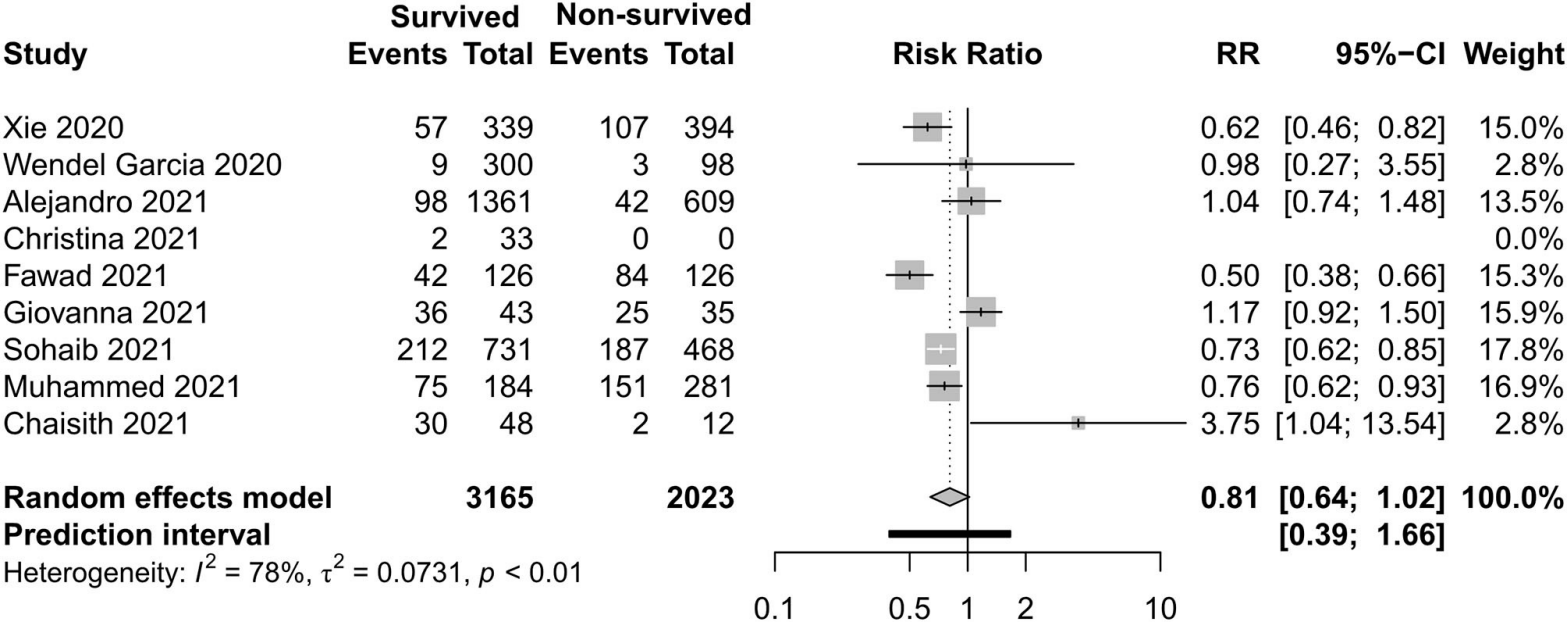
Risk ratio of non-invasive ventilation between survived and non-survived patients.

**Figure 10:**
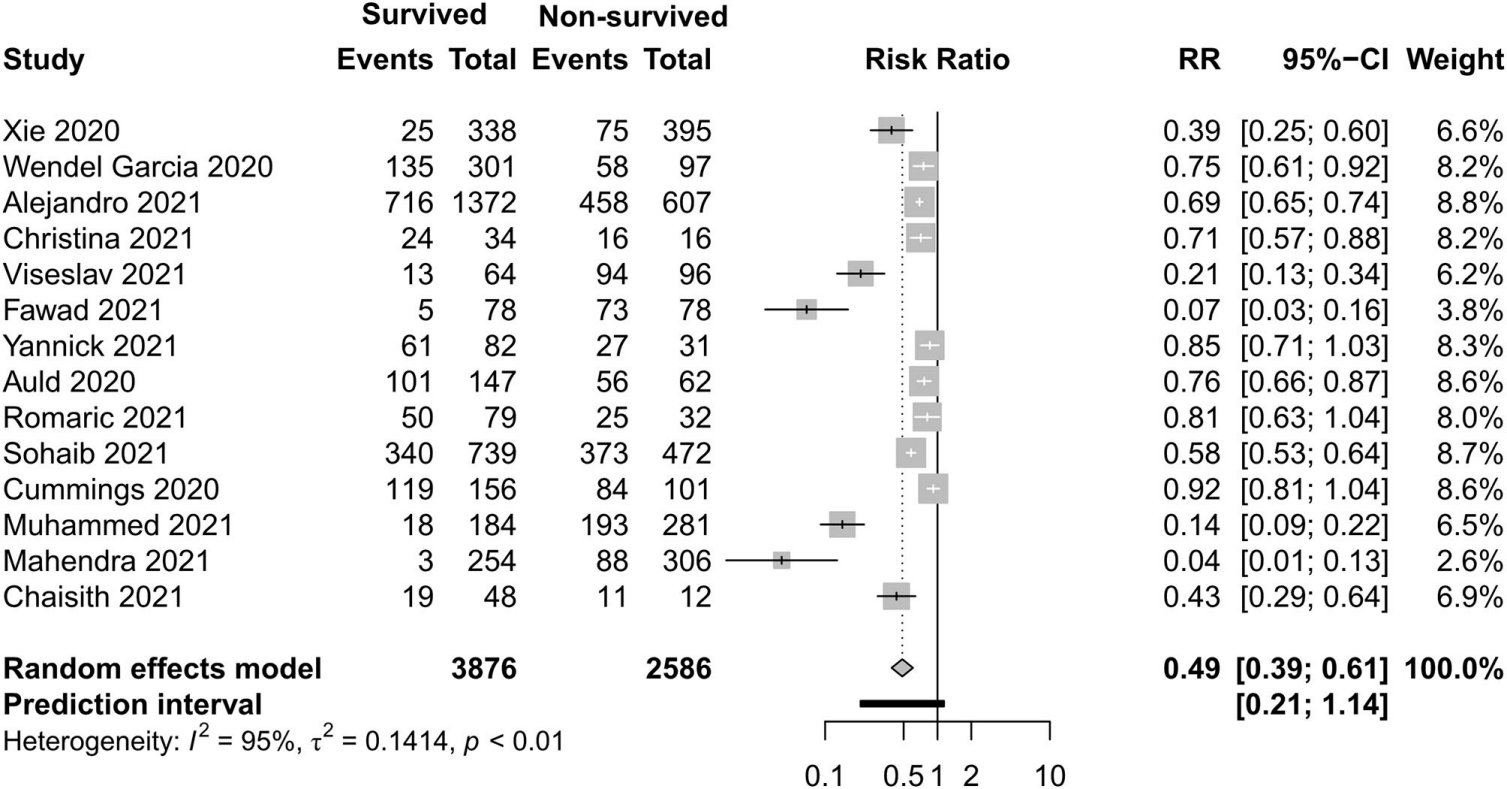
Risk ratio of invasive mechanical ventilation between survived and non-survived patients.

**Figure 11:**
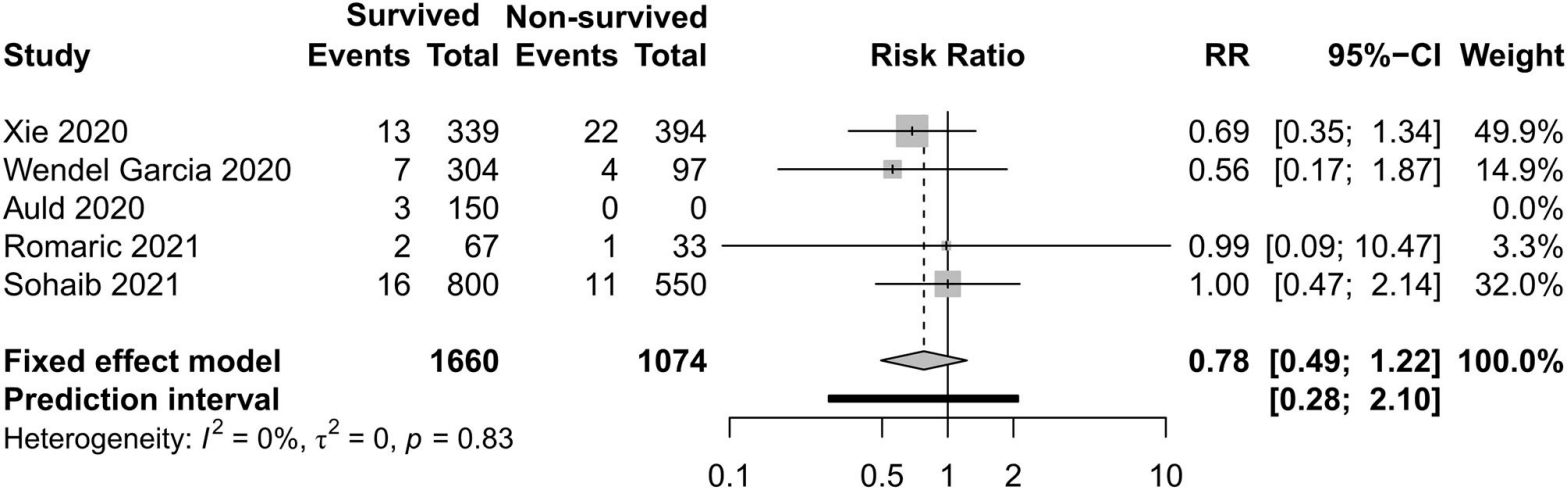
Risk ratio of extracorporeal membrane oxygenation between survived and non-survived patients.

Figures 8–11 display different ways of respiratory support care during ICU hospitalization between surviving and non-surviving patients. High-flow nasal oxygenation (HFNO) was more commonly used in non-surviving patients [with RR 1.33, 95% CI (1.13–1.57)], and IMV was more commonly used in surviving patients [with RR 0.49, 95% CI (0.39–0.61)]. There was no statistically significant difference in NIV [RR 0.81, 95% CI (0.64–1.02)] and ECMO [RR 0.78, 95% CI (0.49–1.22)] between the two groups.

### Renal and Cardiac Support Care

**Figure 12:**
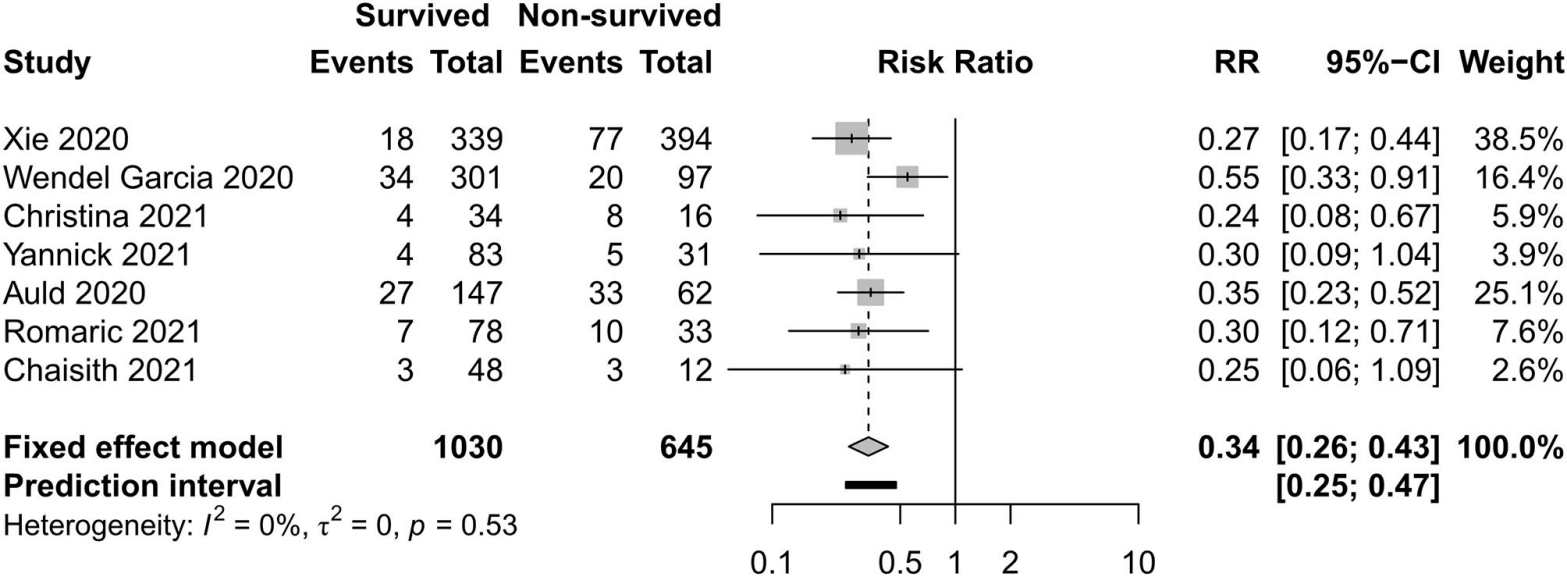
Risk ratio of kidney replacement therapy between survived and non-survived patients.

**Figure 13:**
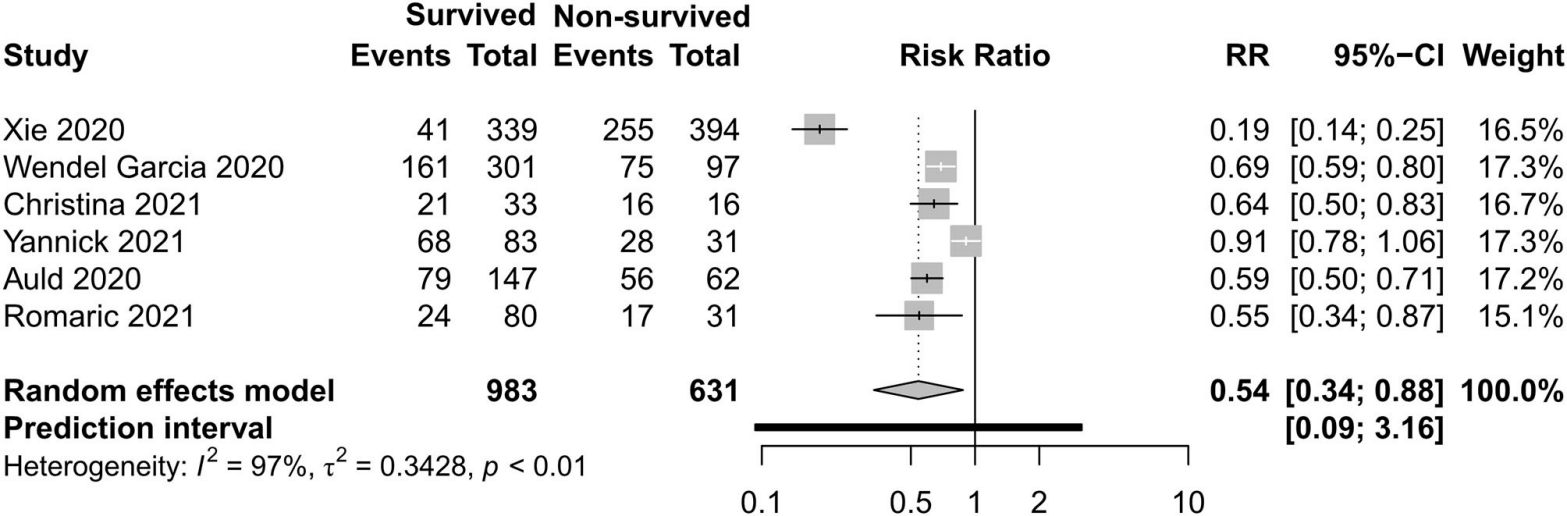
Risk ratio of vasopressors between survived and non-survived patients.

Figures 12, 13 exhibit the surviving patients who received more KRT [RR 0.34, 95% CI (0.26–0.43)] and vasopressor [RR 0.54, 95% CI (0.34–0.88)].

### Quality of Evidence

We evaluated the quality of evidence for 11 outcomes. Among them, two outcomes (18%) were graded as of moderate quality, four outcomes (36%) were graded as of low quality, and five (45%) outcomes were graded as of very low quality. We produced “GRADE evidence profiles,” and the details of GRADE can be found in Supplementary File 5.

### Sensitivity Analysis

We conducted a sensitivity analysis on each result by omitting one study at a time. No study had a significant impact on the results of the meta-analysis (Supplementary File 6). A sensitivity analysis showed that all studies had little or acceptable effect on the total combined effect and that the results were stable.

## Discussion

The epidemic of COVID-19 is not stopping yet, especially in western countries. In previous reports, the incidence of mortality associated with critically ill patients remains poorly characterized. The novel findings in this study include the mortality of critically ill patients with laboratory-confirmed COVID-19 worldwide and the clinical interventions between surviving and non-surviving patients. The results show that all-cause mortality in ICU was 35% and mortality in hospital was 32% around the world for the year 2020. Differences were distinct between regions. The incidence of mortality that occurred in Southeast Asia was as high as 48%, followed by 39% in China and the Middle East. The lowest incidence occurred in America, which is 15%. The plausible explanations for the high mortality in China and other Asia countries are that the arrival and peak of the COVID-19 pandemic in Asia were earlier than in any region, and there was a shortage of ICU resources and experience. Moreover, data may be subject to patient selection for ICU admission, and some nations adopted a stringent strategy (19). In addition, mortality also relates to the time of follow-up. Some of the participants remained in the hospital in mechanical ventilation even at the end of follow-up. A recent meta-analysis reported that all-cause mortality associated with COVID-19 was 10% overall and 34% in patients admitted to the ICU (7), but most of their participants were from China; in this part, we had a close result. This new meta-analysis included more participants and covered much wider regions.

Early identification and prompt organ function support care would provide relief in critical cases (53). Among the included studies, five identified independent risk factors were associated with ICU mortality from laboratory parameters to clinical intervention, but the results are not the same (22, 25, 38, 50, 51, 54). We compared the baseline clinical characteristics between surviving and non-surviving patients. What we found based on the univariate analysis was that old age, APACHEII score, and SOFA score displayed consistency with multivariate Cox regression analysis in these five studies. Besides these, the PaO_2_/FiO_2_ ratio is an important index to reflect the severity of respiratory failure. Our results also showed that the PaO_2_/FiO_2_ ratio is helpful to predict the outcome.

With regard to the outcome of the clinical interventions of this meta-analysis, respiratory support is the most important part of life sustaining treatments. According to this study, HFNO during ICU hospitalization was more often used in non-surviving patients, and IMV was more often used in surviving patients. In previous studies, Auld and Capone (22, 54) reported that receipt of IMV was associated with a decreased likelihood of survival. When we discuss the difference of respiratory support, respiratory support as rescue therapy and the different severity levels of the two groups should not be ignored. HFNO and NIV can be safely used in COVID-19-related mild–moderate ARDS. In the study of non-COVID-19, HFNO has been associated with lower mortality in hypoxemic respiratory failure (55), but in some moderate–severe ARDS patients, HFNO or NIV should be used cautiously due to rapid progression to severe type and a high risk of treatment failure. According to Mukhtar et al. (56), the use of NIV with a predefined algorithm in subjects with moderate–severe COVID-19 ARDS was successful in 77% of the subjects. IMV is the most widely used therapy of severe hypoxemia. The population with IMV was larger than with non-invasive support in this study. The need of endotracheal intubation and invasive mechanical ventilation was eight times that of non-invasive ventilation in a previous study (30). Although the timing of IMV is disputed, as evidenced in a recent publication, a meta-analysis reported that early intubation was not associated with improved survival (57). A latest meta-analysis (42) reported that the timing of intubation may have not influenced the mortality of critically ill patients with COVID-19. ECMO can be taken into consideration if the respiratory dysfunction of patients develop into severe ARDS, which cannot sustain with IMV, but this salvage treatment did not have a statistically significant difference between the two groups. In a study with a small sample (3), two of five patients survived by the support of ECMO. The appropriate time and eligible patients need to be evaluated.

In a previous research, as high as 31% of patients in a cohort developed severe acute kidney injury requiring renal replacement therapy during hospitalization (25). High creatinine level, AKI, and receipt of RRT were independent risk factors for the in-hospital mortality of patients (22, 51, 58). Similarly, high high-sensitivity cardiac troponin I level, ischemic heart disease, cardiac injury, and vasopressor support were associated with death in patients (22, 38, 50, 51, 54). In the present study, the result shows that vasopressors and RRT were more often used in the surviving group.

There were some limitations in the current study that must be acknowledged. First is the high level of heterogeneity in the study. Plausible explanations for the heterogeneous risks of mortality include differences in age, nation and race, disease severity, and insufficient length of follow-up. It was difficult for us to control for the effects of these confounding factors. The heterogeneity in the component studies was addressed with random-effects models. Second, as for the secondary outcomes, is that this part of the clinical interventions was derived from an observational cohort, not a randomized controlled trial, so these results should be treated cautiously. The key purpose of this study is to describe the effect of the actual use of various clinical interventions in the surviving group and non-surviving group rather than the impact of individual measures on the prognosis. Third is that most studies were retrospective and recall bias might have occurred.

## Conclusions

Mortality was high in critically ill patients with COVID-19 based on low-quality evidence, and intercontinental differences existed. The early identification of critical characteristics and the use of support care help to indicate the outcome of critically ill patients.

## Data Availability Statement

The datasets presented in this study can be found in online repositories. The names of the repository/repositories and accession number(s) can be found in the article/Supplementary Material.

## Author Contributions

LL, YC, and ZQ: concept and design. LL: administrative, technical, or material support. ZQ and SL: statistical analysis and drafting of the manuscript. LL and YC: supervision. All authors critical revision of the manuscript for important intellectual content, acquisition, analysis, or interpretation of data.

## Conflict of Interest

The authors declare that the research was conducted in the absence of any commercial or financial relationships that could be construed as a potential conflict of interest.

## Publisher's Note

All claims expressed in this article are solely those of the authors and do not necessarily represent those of their affiliated organizations, or those of the publisher, the editors and the reviewers. Any product that may be evaluated in this article, or claim that may be made by its manufacturer, is not guaranteed or endorsed by the publisher.
