# Supplementary material for: Mortality and Clinical Interventions in Critically ill Patient With Coronavirus Disease 2019: A Systematic Review and Meta-Analysis
Source: Front Med (Lausanne). 2021 Jul 23;8:635560. doi: 10.3389/fmed.2021.635560 (PMC8342953; doi:10.3389/fmed.2021.635560)
Supplement: Supplementary file 1 [file Data_Sheet_1.ZIP › Supplementary Material/Supplement 1. Search Strategies.docx]

Search Strategies

Search Time: 2021/5/15

PubMed (n=7438)

#1 "COVID-19"[Supplementary Concept]

#2 "Severe Acute Respiratory Syndrome Coronavirus 2"[Supplementary Concept]

#3 "SARS Virus"[Mesh]

#4 "COVID-19"[Title/Abstract]

#5 "SARS-COV-2"[Title/Abstract]

#6 "Novel coronavirus" [Title/Abstract]

#7 “2019-novel coronavirus” [Title/Abstract]

#8 “coronavirus disease-19” [Title/Abstract]

#9 “coronavirus disease 2019” [Title/Abstract]

#10 “Novel CoV” [Title/Abstract]

#11 “2019-nCoV” [Title/Abstract]

#12 “2019-CoV” [Title/Abstract]

#13 OR/#1-#12

#14 “critically ill patient” [Title/Abstract]

#15 “severely ill patient” [Title/Abstract]

#16 /#14 OR #15

#17 #13 AND #16

#18 “mortality” [Title/Abstract]

#19 #17 AND #18

#20 " mechanical ventilation "[Title/Abstract]

#21 " invasive mechanical ventilation "[Title/Abstract]

#22 " high flow nasal cannula "[Title/Abstract]

#23 " non-invasive ventilation " [Title/Abstract]

#24 “extracorporeal membrane oxygenation” [Title/Abstract]

#25 “renal replacement therapy” [Title/Abstract]

#26 “kidney replacement therapy” [Title/Abstract]

#27 “vasopressor” [Title/Abstract]

#28 OR/#20-#27

#29 #19 AND #28
