# Supplementary material for: Mortality and Clinical Interventions in Critically ill Patient With Coronavirus Disease 2019: A Systematic Review and Meta-Analysis
Source: Front Med (Lausanne). 2021 Jul 23;8:635560. doi: 10.3389/fmed.2021.635560 (PMC8342953; doi:10.3389/fmed.2021.635560)
Supplement: Supplementary file 1 [file Data_Sheet_1.ZIP › Supplementary Material/Supplement 2-2. bias in case series studies.docx]

Supplement 2-2. Assessment of risk bias in case series

| Study | Were there clear criteria for inclusion in the case series? | Was the condition measured in a standard, reliable way for all participants included in the case series? | Were valid methods used for identification of the condition for all participants included in the case series? | Did the case series have consecutive inclusion of participants? | Did the case series have complete inclusion of participants? | Was there clear reporting of the demographics of the participants in the study? | Was there clear reporting of clinical information of the participants? | Were the outcomes or follow up results of cases clearly reported? | Was there clear reporting of the presenting site(s)/clinic(s) demographic information? | Was statistical analysis appropriate? | JBI  Score |
| --- | --- | --- | --- | --- | --- | --- | --- | --- | --- | --- | --- |
| Xie 2020 | Yes | No | Yes | Yes | Yes | Yes | Yes | Yes | No | Yes | 8 |
| Mitra 2020 | Yes | Yes | No | Yes | Yes | Yes | Yes | Yes | No | Yes | 8 |
| Aleva 2020 | Yes | Yes | Yes | Yes | No | Yes | Yes | Yes | No | Yes | 8 |
| Hu 2020 | Yes | Yes | Yes | Yes | No | Yes | Yes | Yes | Yes | Yes | 9 |
| Fawad 2021 | Yes | Yes | Yes | Yes | No | Yes | Yes | Yes | No | Yes | 8 |
| Li 2021 | Yes | No | Yes | No | No | Yes | Yes | Yes | No | Yes | 6 |
