# Supplementary material for: Mortality and Clinical Interventions in Critically ill Patient With Coronavirus Disease 2019: A Systematic Review and Meta-Analysis
Source: Front Med (Lausanne). 2021 Jul 23;8:635560. doi: 10.3389/fmed.2021.635560 (PMC8342953; doi:10.3389/fmed.2021.635560)
Supplement: Supplementary file 1 [file Data_Sheet_1.ZIP › Supplementary Material/Supplement 3-1. bias in cohort studies.docx]

Supplement 2-1 . Assessment of risk bias in cohort studies

| Study | Type | Selection | | | | Comparability | Outcome | | | NOS score |
| --- | --- | --- | --- | --- | --- | --- | --- | --- | --- | --- |
|  |  | Representativeness of the exposed cohort | Selection of the non-exposed cohort | Ascertainment of exposure | Demonstration that outcome of interest was not present at start of study | Comparability of cohorts on the basis of the design or analysis | Assessment of outcome | Was follow-up long enough for outcomes to occur | Adequacy of follow up of cohorts |  |
| Wendel Garcia 2020 | Cohort | 1 | 1 | 1 | 1 | 1 | 1 | 0 | 0 | 6 |
| Li 2020 | Cohort | 1 | 1 | 1 | 0 | 1 | 1 | 1 | 0 | 6 |
| Sandra 2021 | Cohort | 1 | 1 | 1 | 0 | 1 | 1 | 0 | 1 | 6 |
| Chen 2021 | Cohort | 1 | 0 | 1 | 0 | 1 | 1 | 0 | 0 | 4 |
| Abdulrahman 2021 | Cohort | 1 | 1 | 1 | 0 | 1 | 1 | 0 | 0 | 5 |
| Alejandro 2021 | Cohort | 1 | 1 | 1 | 1 | 1 | 0 | 1 | 1 | 7 |
| Christina 2021 | Cohort | 1 | 1 | 1 | 1 | 1 | 1 | 1 | 0 | 7 |
| Viseslav 2021 | Cohort | 1 | 1 | 1 | 0 | 0 | 1 | 0 | 1 | 5 |
| Gamberini 2020 | Cohort | 1 | 1 | 1 | 1 | 1 | 1 | 0 | 0 | 6 |
| Giovanna 2021 | Cohort | 1 | 1 | 1 | 0 | 1 | 1 | 0 | 1 | 6 |
| Guillaume 2021 | Cohort | 1 | 1 | 1 | 0 | 2 | 0 | 0 | 1 | 6 |
| Josef 2021 | Cohort | 1 | 1 | 1 | 1 | 1 | 1 | 0 | 0 | 6 |
| Pedro 2021 | Cohort | 1 | 1 | 1 | 1 | 2 | 1 | 1 | 0 | 8 |
| Ramazan 2021 | Cohort | 1 | 0 | 0 | 0 | 1 | 1 | 0 | 1 | 4 |
| Shruti 2021 | Cohort | 1 | 1 | 1 | 0 | 2 | 1 | 1 | 0 | 7 |
| Yannick 2021 | Cohort | 1 | 1 | 0 | 0 | 1 | 0 | 0 | 0 | 3 |
| Auld 2020 | Cohort | 1 | 1 | 1 | 1 | 1 | 0 | 1 | 0 | 6 |
| Cumming 2020 | Cohort | 1 | 1 | 1 | 0 | 1 | 1 | 0 | 1 | 6 |
| Romaric 2021 | Cohort | 1 | 1 | 1 | 1 | 2 | 1 | 1 | 0 | 8 |
| Sohaib 2021 | Cohort | 1 | 1 | 1 | 0 | 1 | 1 | 0 | 0 | 5 |
| Osaid 2021 | Cohort | 1 | 1 | 1 | 1 | 2 | 1 | 1 | 1 | 9 |
| Muhammed 2021 | Cohort | 1 | 1 | 1 | 1 | 1 | 1 | 1 | 1 | 8 |
| Mahendra 2021 | Cohort | 1 | 1 | 0 | 0 | 2 | 0 | 1 | 1 | 6 |
| Arshia 2021 | Cohort | 1 | 1 | 0 | 0 | 1 | 1 | 0 | 0 | 4 |
| Chaisith 2021 | Cohort | 1 | 1 | 0 | 1 | 1 | 1 | 0 | 0 | 5 |
| Mostafa 2021 | Cohort | 1 | 1 | 1 | 1 | 1 | 1 | 1 | 1 | 8 |

a Score based on the Newcastle−Ottawa scale guidelines for cohort studies and JBI Score for case series
