# Supplementary material for: Mortality and Clinical Interventions in Critically ill Patient With Coronavirus Disease 2019: A Systematic Review and Meta-Analysis
Source: Front Med (Lausanne). 2021 Jul 23;8:635560. doi: 10.3389/fmed.2021.635560 (PMC8342953; doi:10.3389/fmed.2021.635560)
Supplement: Supplementary file 1 [file Data_Sheet_1.ZIP › Supplementary Material/Supplement 3-Table1.docx]

**Table 1. Basic characteristics of included studies of mortality of critically ill patients**

| Region | Nation | Study | No. patients | Study design | Single- or multi-center | Date | Follow-up | outcome |
| --- | --- | --- | --- | --- | --- | --- | --- | --- |
| Asia | China | Xie 2020 | 733 | retrospective case series | multi-center | Jan. 1 to Feb. 29 | 28-day | 394 patients died |
|  | China | Li 2020 | 268 | retrospective, cohort study | single-center | Jan. 26 to Feb. 5 | 32-day | 87 patients died, 85 discharged from hospital |
|  | China | Hu 2020 | 55 | retrospective case series | single-center | Jan. 8 to Mar. 12 | 28-day | 16 patients died, 33 discharged home.6 transferred to isolation wards |
|  | China | Chen 2021 | 192 | retrospective case series | single-center | Jan.28 to Mar.13 | until Mar.13 | 50 died in the hospital and 142 were discharged |
|  | China | Li 2021 | 123 | retrospective observational study | single-center | Feb.9 to Apr.6 | until Apr.6 | 57 died in ICU hospitalization and 66 were discharged |
|  | Pakistan | Fawad 2021 | 204 | cross-sectional study | single-center | Apr.1 to Aug.31 | until Aug.31 | 157 died in ICU hospitalization and 47 shifting from the ICU to a general isolation ward |
|  | India | Mahendra 2021 | 560 | retrospective observational study | single-center | Jun.1 to Oct.30 | 30-day | 306 died in hospital |
|  | Thailand | Chaisith 2021 | 60 | prospective observational study | single-center | Jan. 1 to 31 | until Jan. 31 | 12 died and 48 patients were alive at ICU discharge. |
| Middle East | Qatar | Mostafa 2021 | 60 | prospective observational study | single-center | Jun.26 to Aug.5 | 60-day or died or discharged from the ICU | 7 died |
|  | Iran | Arshia 2021 | 133 | retrospective cohort study | single-center | Feb.1. to Jun.30 | until Jun.30 | 77 died in ICUs and other patients were recovered or discharged from ICUs. |
|  | Saudi Arabia | Abdulrahman 2021 | 352 | retrospective observational study | single-center | Mar.20 to May.31 | 28-day | 113 died in ICU hospitalization |
|  | Libya | Muhammed 2021 | 465 | prospective cohort study | multi-center | May.29 to Dec.30 | 60-day | 281 died in ICU and 184 discharged alive |
| Europe | Europe | Wendel Garcia 2020 | 398 | prospective cohort study | multi-center | Mar. 13 to Apr. 22 | 40-day | 97 patients died and 301 discharged |
|  | France | Guillaume 2021 | 14351 | retrospective cohort study | multi-center | Feb.1 to Jun.9 | until Jun.9 | 3790 died in hospital |
|  | Spain | Alejandro 2021 | 2022 | prospective observational | multi-center | Feb.22 to May.11 | 90-day | 660 died in ICU and 1362 discharged from ICU |
|  | Italy | Gamberini 2020 | 391 | retrospective observational study | multi-center | Feb.22 to May.4 | until May 15 | 141 died in ICU hospitalization,39 still in ICU |
|  | Italy | Giovanna 2021 | 78 | retrospective observational study | single-center | Mar.11.to Apr.27 | until Apr.27 | 35 patients died during hospitalization, 43 discharge from the ICU |
|  | Italy | Grasselli2020 | 1581 | retrospective case series | multi-center | Feb. 20 to Mar. 18 | 7-day | 405 patients died, 920 still in the ICU, 256 discharged |
|  | Sweden | Sandra 2021 | 152 | retrospective observational study | single-center | Mar.1 to Apr.31 | 28-day | 43 died in ICU |
|  | Sweden | Josef 2021 | 92 | prospective observational | single-center | Mar.1 to Jun.30 | 30-day | 21 died in ICU |
|  | Netherland | Aleva 2020 | 50 | retrospective case series | single-center | Mar. 9 to Apr. 7 | 86-day | 13 patients died, 37 survived and discharged from ICU |
|  | Netherland | Yannick 2021 | 114 | retrospective observational study | single-center | Mar.1 to Jun.4 | 28-day | 31 died in ICU hospitalization |
|  | Turkey | Ramazan 2021 | 209 | retrospective observational study | single-center | Mar.24 to Jul.6 | until their outcomes | 82 died in ICU hospitalization |
|  | Serbia | Viseslav 2021 | 160 | retrospective observational study | single-center | Jun.23 to Oct.2 | until their outcomes | 96 died in ICU hospitalization，64 lived |
|  | Greece | Christina 2021 | 50 | prospective observational study | single-center | Mar.11 to Apr.27 | until Apr.27 | 16 patients died, 1 still in the ICU, 33 discharged |
| America | US | Shruti 2021 | 3924 | retrospective cohort study | multi-center | Mar.4 to May.10 | 30-day | 1544 patients died , 2058 discharged alive，322 remained hospitalized. |
|  | Canada | Mitra 2020 | 117 | retrospective case series | multi-center | Feb. 21 to Apr. 14 | 21-day | 18 patients died, 12 remained in ICU, 16 discharged from ICU but remained in hospital, and 71discharged home. |
|  | Brazil | Pedro 2021 | 13301 | retrospective cohort study | multi-center | Feb.27. to Oct.28 | 60-day | 1785 patients died during hospitalization，82 remained hospitalized |
