# Supplementary material for: Mortality and Clinical Interventions in Critically ill Patient With Coronavirus Disease 2019: A Systematic Review and Meta-Analysis
Source: Front Med (Lausanne). 2021 Jul 23;8:635560. doi: 10.3389/fmed.2021.635560 (PMC8342953; doi:10.3389/fmed.2021.635560)
Supplement: Supplementary file 1 [file Data_Sheet_1.ZIP › Supplementary Material/Supplement 6.Sensitivity Analysis/ECMO.pdf]

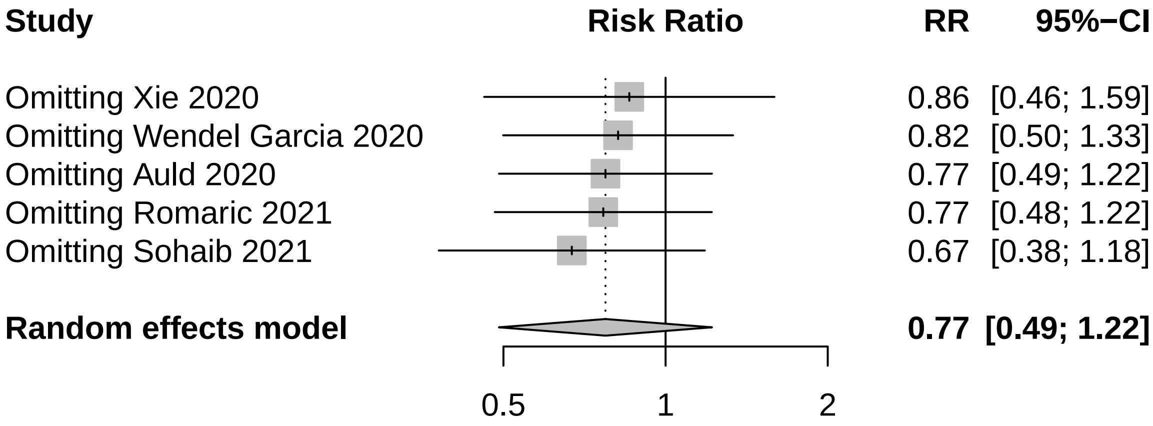

Study
Risk Ratio
RR
95%−CI
Omitting Xie 2020
0.86
[0.46; 1.59]
Omitting Wendel Garcia 2020
0.82
[0.50; 1.33]
Omitting Auld 2020
0.77
[0.49; 1.22]
Omitting Romaric 2021
0.77
[0.48; 1.22]
Omitting Sohaib 2021
0.67
[0.38; 1.18]
Random effects model
0.77
[0.49; 1.22]
0.5
1
2
