# Supplementary material for: Mortality and Clinical Interventions in Critically ill Patient With Coronavirus Disease 2019: A Systematic Review and Meta-Analysis
Source: Front Med (Lausanne). 2021 Jul 23;8:635560. doi: 10.3389/fmed.2021.635560 (PMC8342953; doi:10.3389/fmed.2021.635560)
Supplement: Supplementary file 1 [file Data_Sheet_1.ZIP › Supplementary Material/Supplement 6.Sensitivity Analysis/PFratio.pdf]

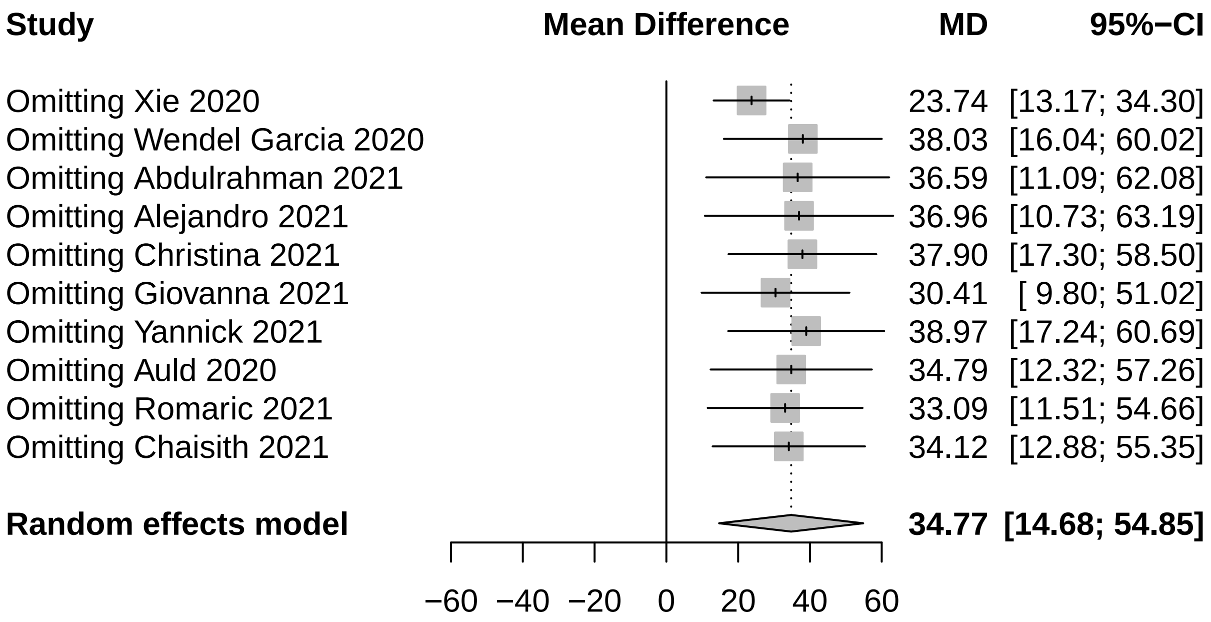
Study
Mean Difference
MD
95%−CI
Omitting Xie 2020 23.74 [13.17; 34.30]
Omitting Wendel Garcia 2020 38.03 [16.04; 60.02]
Omitting Abdulrahman 2021 36.59 [11.09; 62.08]
Omitting Alejandro 2021 36.96 [10.73; 63.19]
Omitting Christina 2021 37.90 [17.30; 58.50]
Omitting Giovanna 2021 30.41 [9.80; 51.02]
Omitting Yannick 2021 38.97 [17.24; 60.69]
Omitting Auld 2020 34.79 [12.32; 57.26]
Omitting Romaric 2021 33.09 [11.51; 54.66]
Omitting Chaisith 2021 34.12 [12.88; 55.35]
Random effects model 34.77 [14.68; 54.85]
−60 −40 −20 0 20 40 60
