# Supplementary figures and images for: Mortality and Clinical Interventions in Critically ill Patient With Coronavirus Disease 2019: A Systematic Review and Meta-Analysis
Source: Front Med (Lausanne). 2021 Jul 23;8:635560. doi: 10.3389/fmed.2021.635560 (PMC8342953; doi:10.3389/fmed.2021.635560)

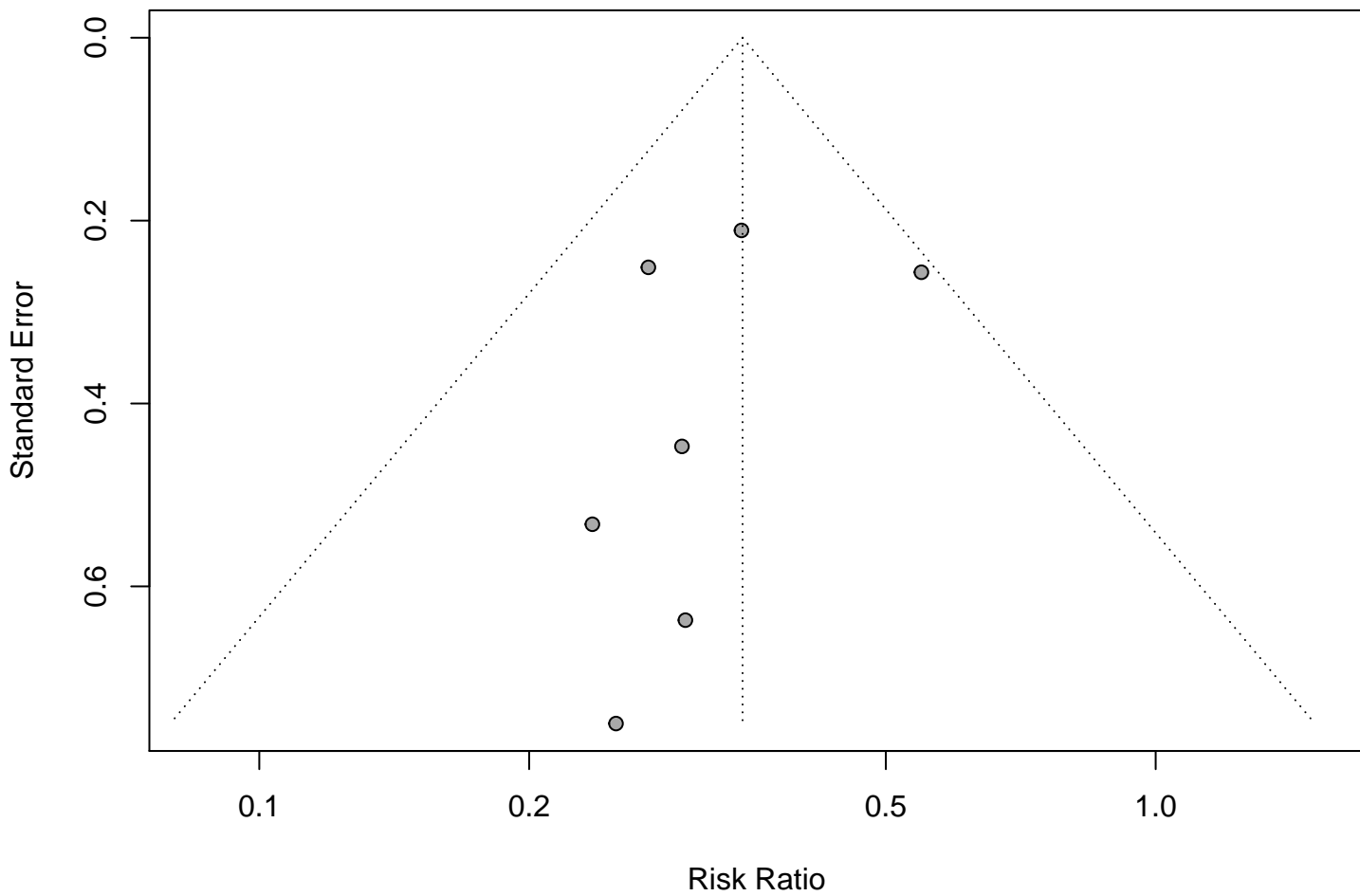

Supplement: Supplementary file 1 [file Data_Sheet_1.ZIP › Supplementary Material/Supplement 4. funnel plot/ICU_motality.pdf]

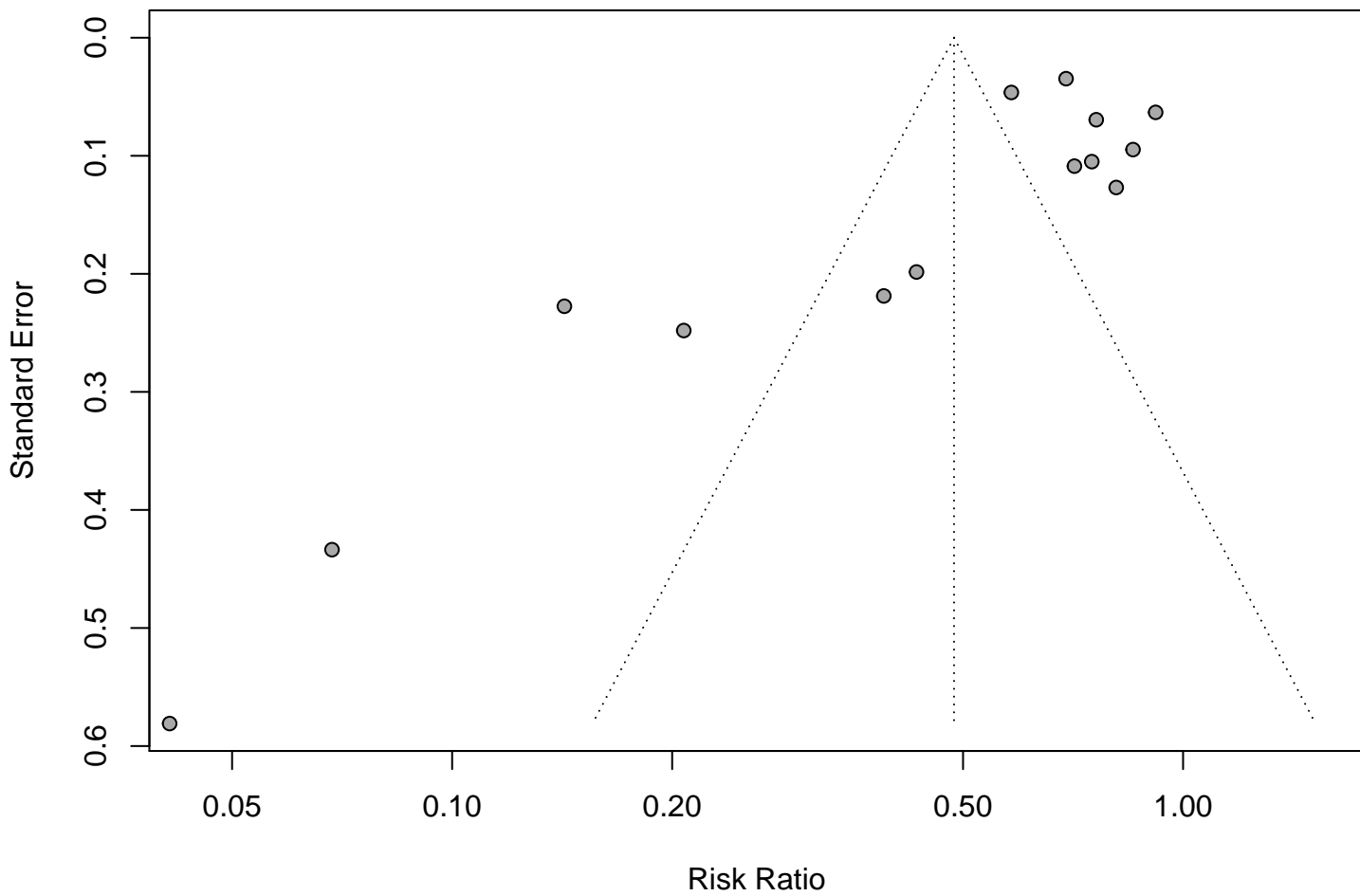

Supplement: Supplementary file 1 [file Data_Sheet_1.ZIP › Supplementary Material/Supplement 4. funnel plot/IMV.pdf]

Standard Error

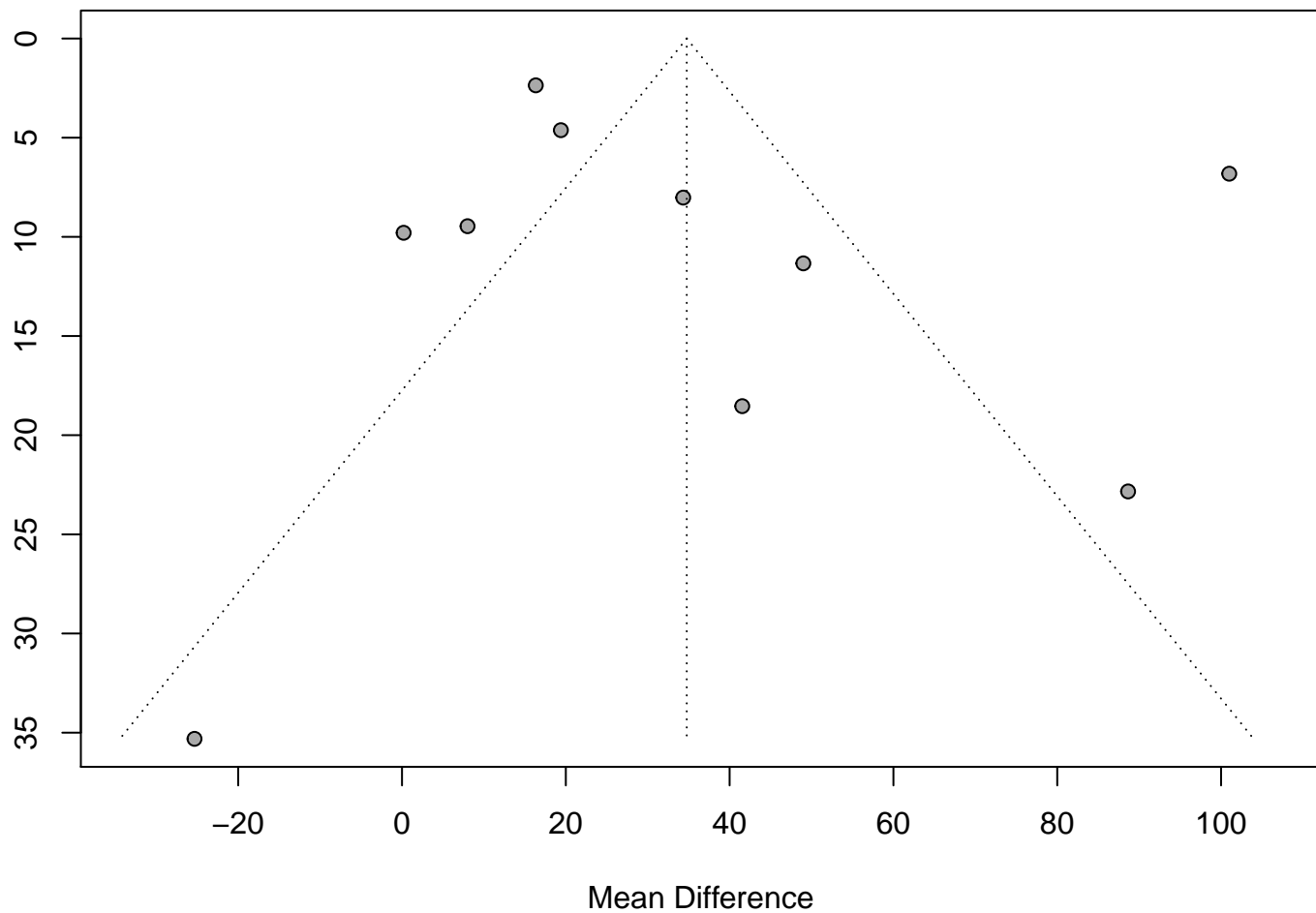

Supplement: Supplementary file 1 [file Data_Sheet_1.ZIP › Supplementary Material/Supplement 4. funnel plot/Pfratio.pdf]

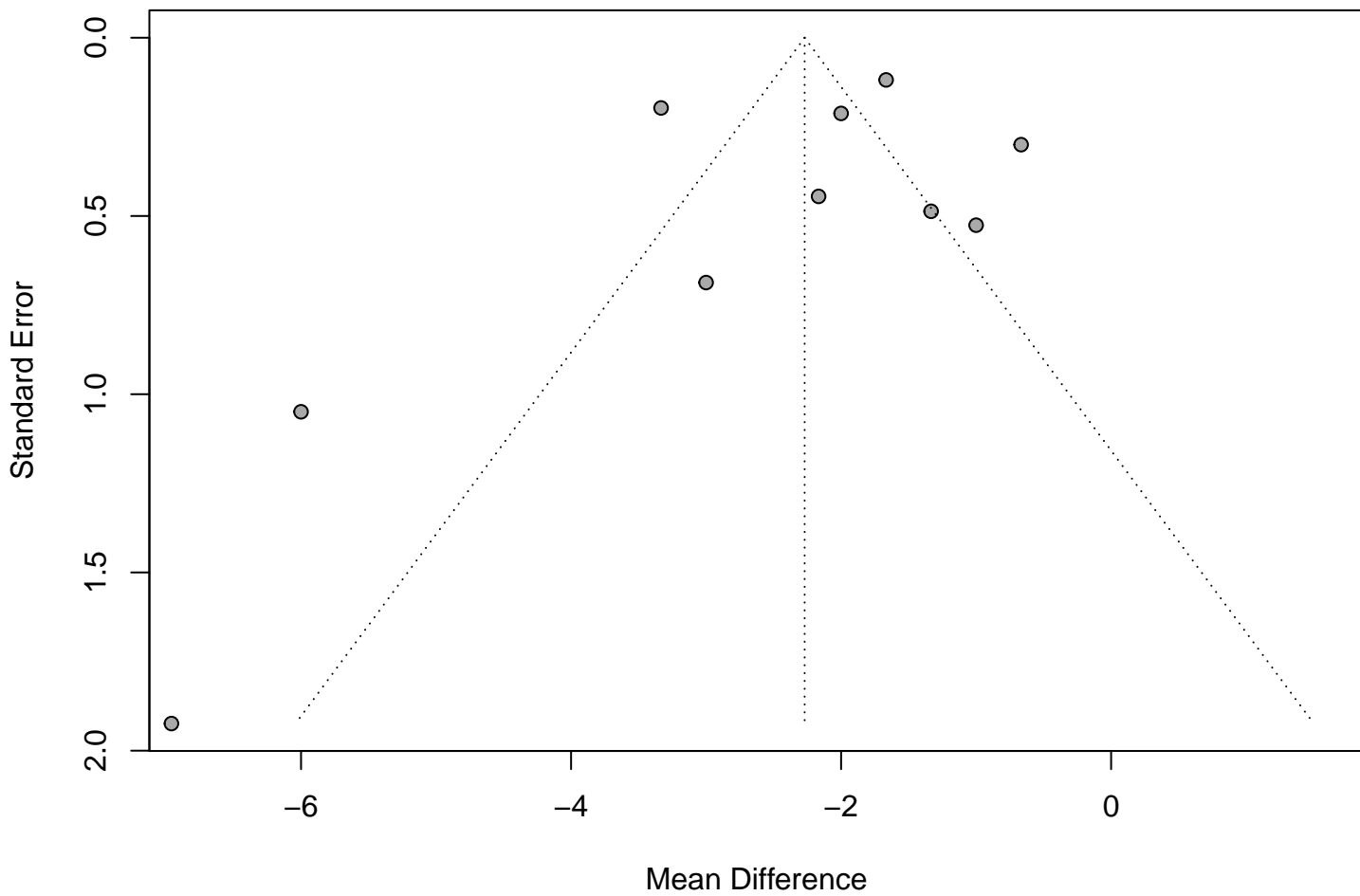

Supplement: Supplementary file 1 [file Data_Sheet_1.ZIP › Supplementary Material/Supplement 4. funnel plot/SOFA.pdf]

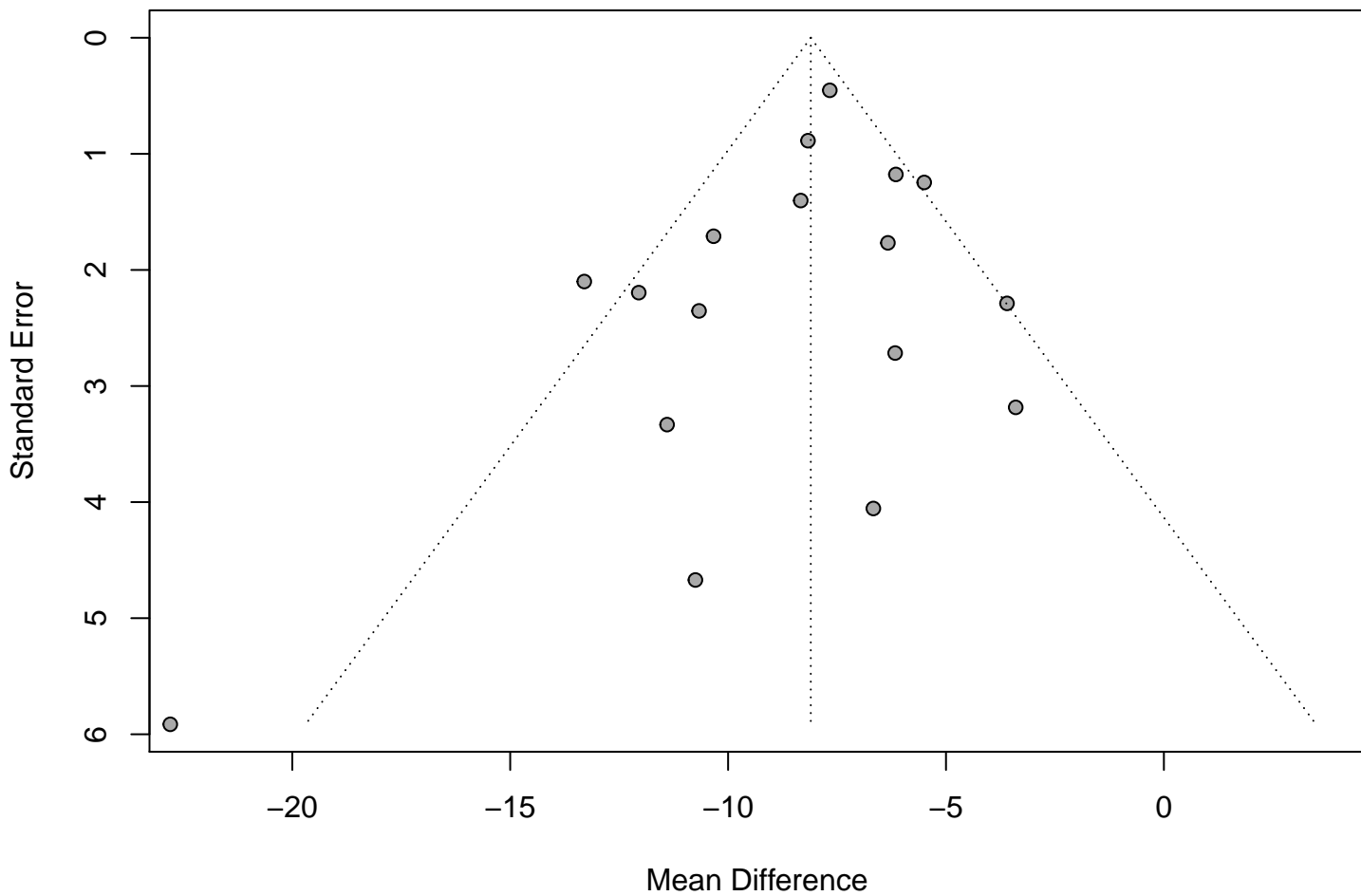

Supplement: Supplementary file 1 [file Data_Sheet_1.ZIP › Supplementary Material/Supplement 4. funnel plot/age.pdf]

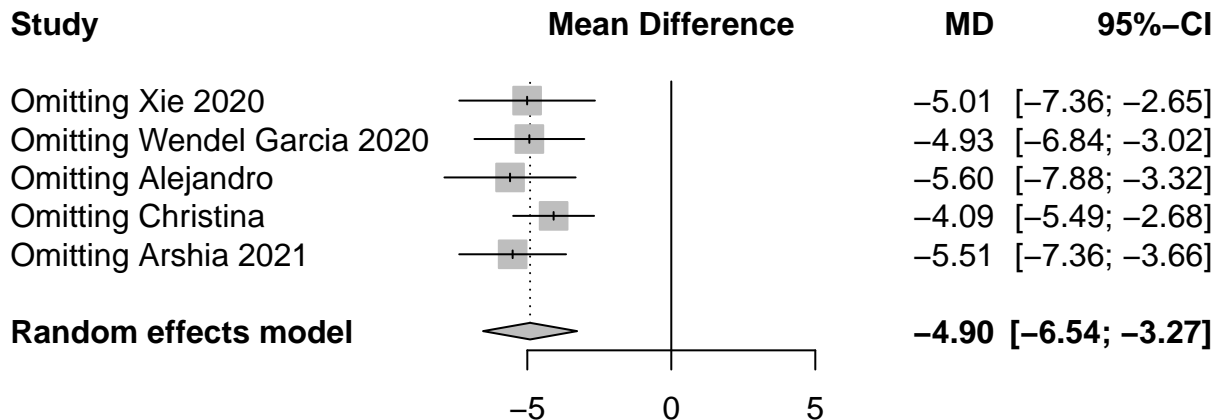

Supplement: Supplementary file 1 [file Data_Sheet_1.ZIP › Supplementary Material/Supplement 6.Sensitivity Analysis/APACHII.pdf]

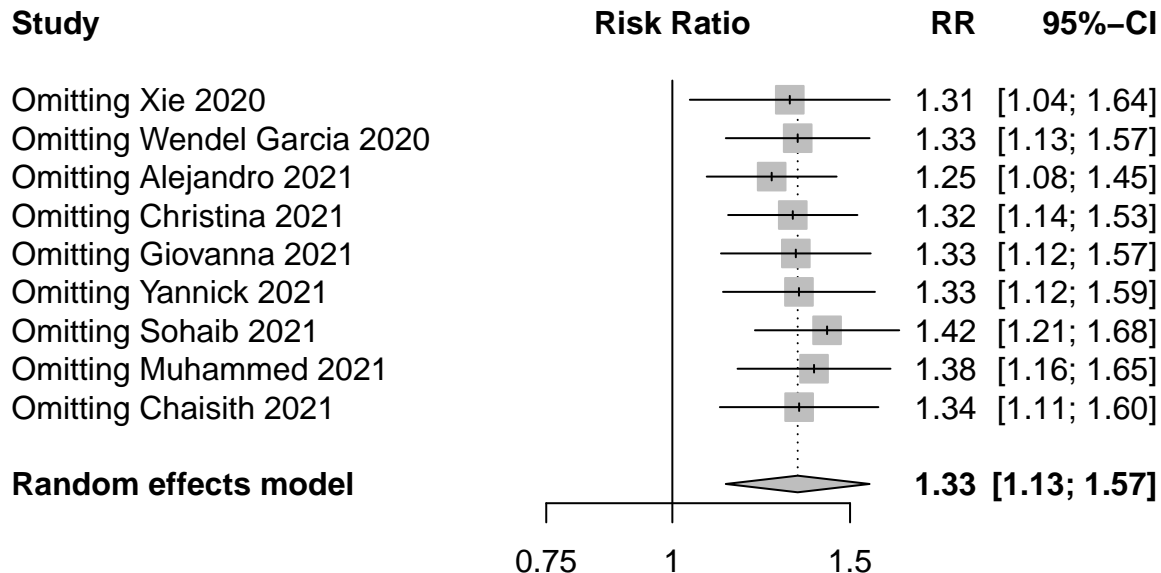

Supplement: Supplementary file 1 [file Data_Sheet_1.ZIP › Supplementary Material/Supplement 6.Sensitivity Analysis/HFNO.pdf]

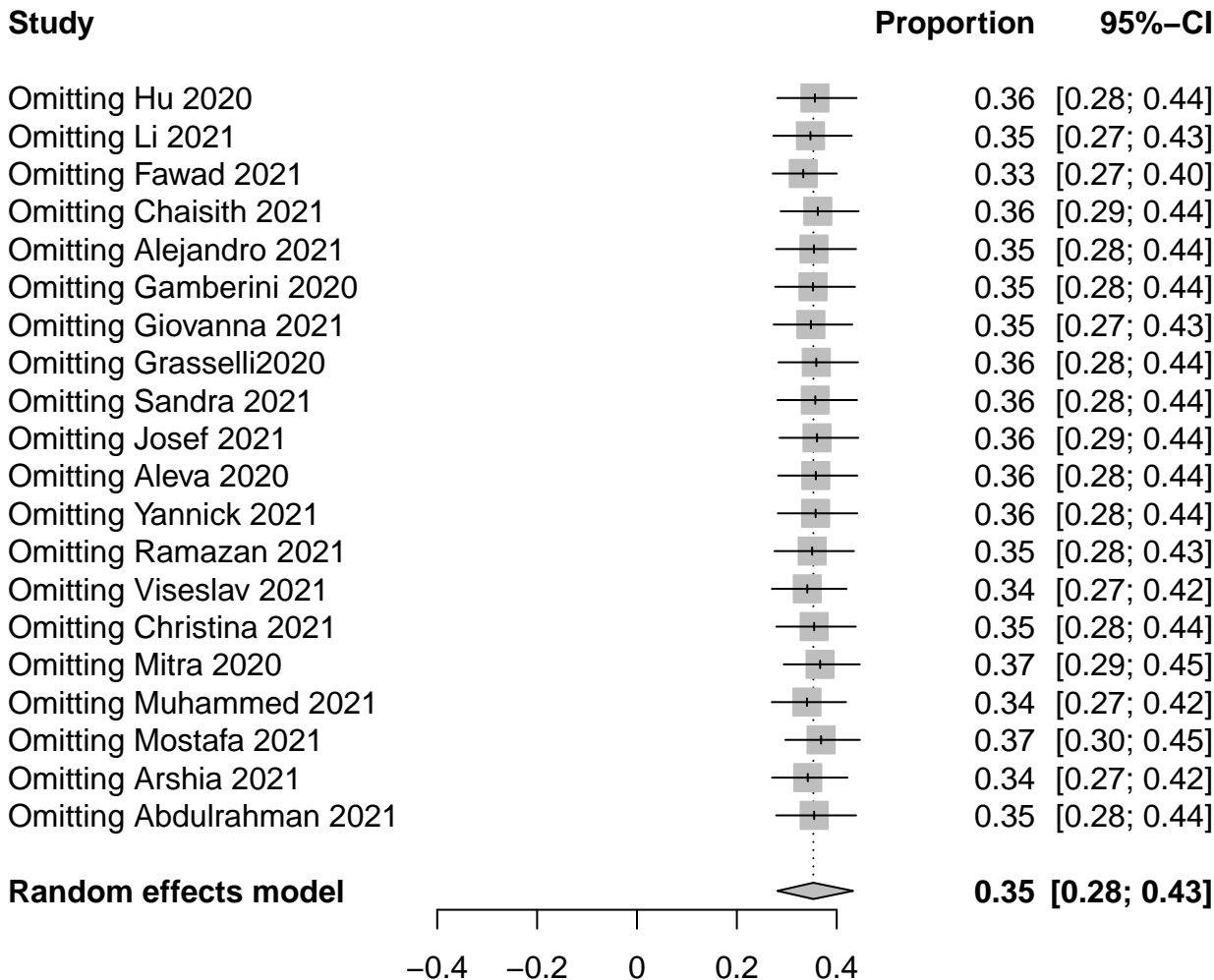

Supplement: Supplementary file 1 [file Data_Sheet_1.ZIP › Supplementary Material/Supplement 6.Sensitivity Analysis/ICU_motality-sen.pdf]

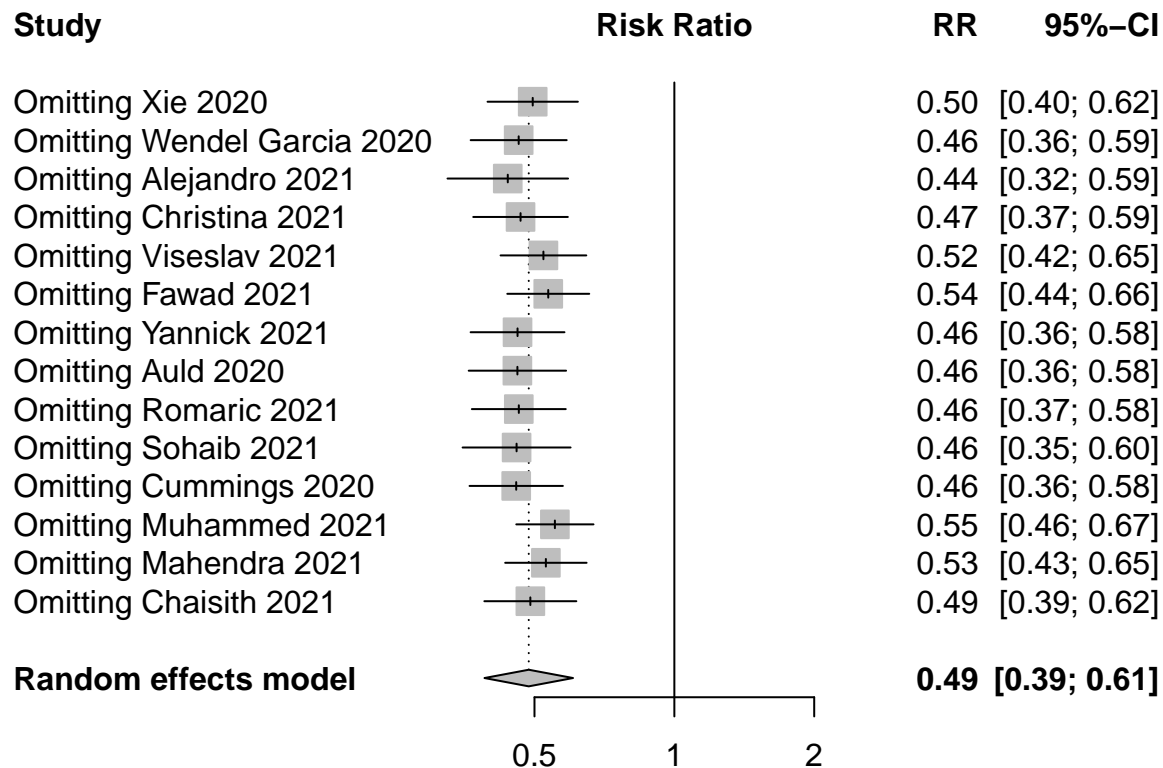

Supplement: Supplementary file 1 [file Data_Sheet_1.ZIP › Supplementary Material/Supplement 6.Sensitivity Analysis/IMV.pdf]

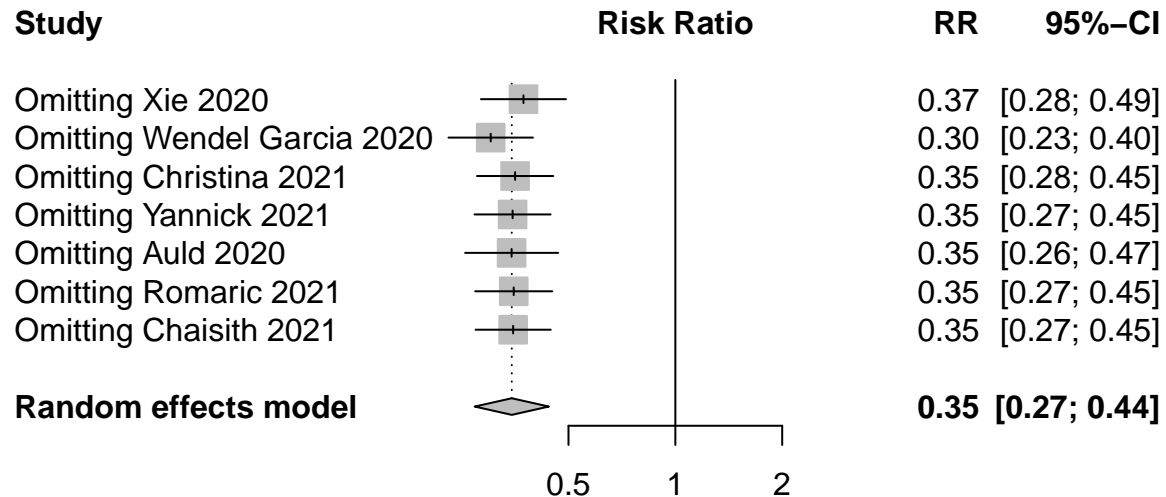

Supplement: Supplementary file 1 [file Data_Sheet_1.ZIP › Supplementary Material/Supplement 6.Sensitivity Analysis/KRT.pdf]

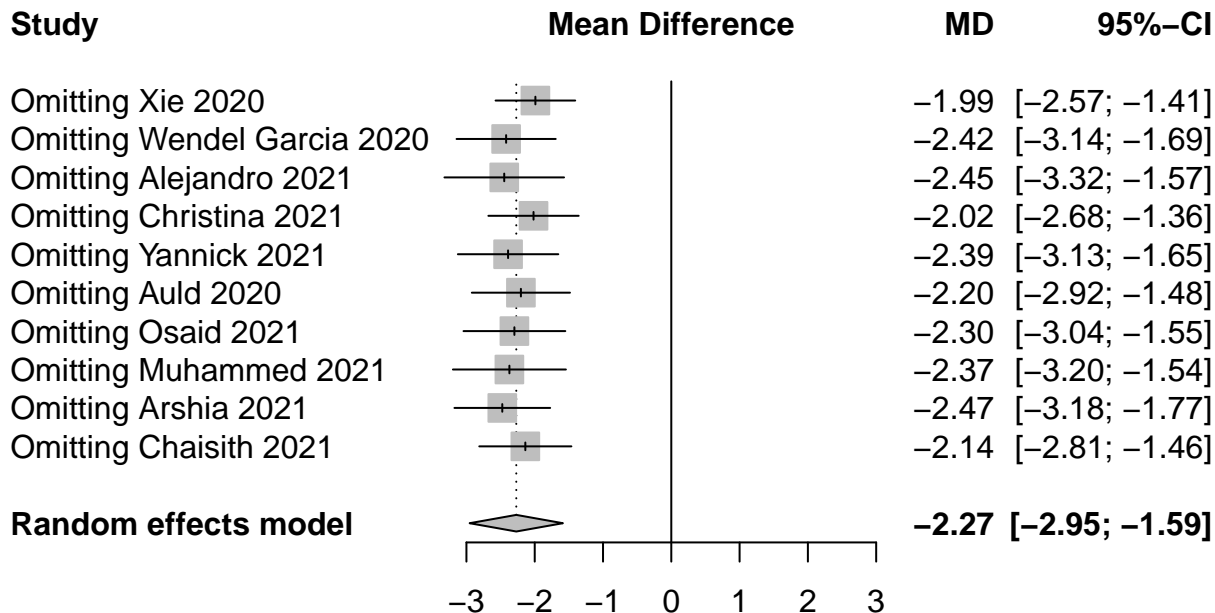

Supplement: Supplementary file 1 [file Data_Sheet_1.ZIP › Supplementary Material/Supplement 6.Sensitivity Analysis/SOFA.pdf]

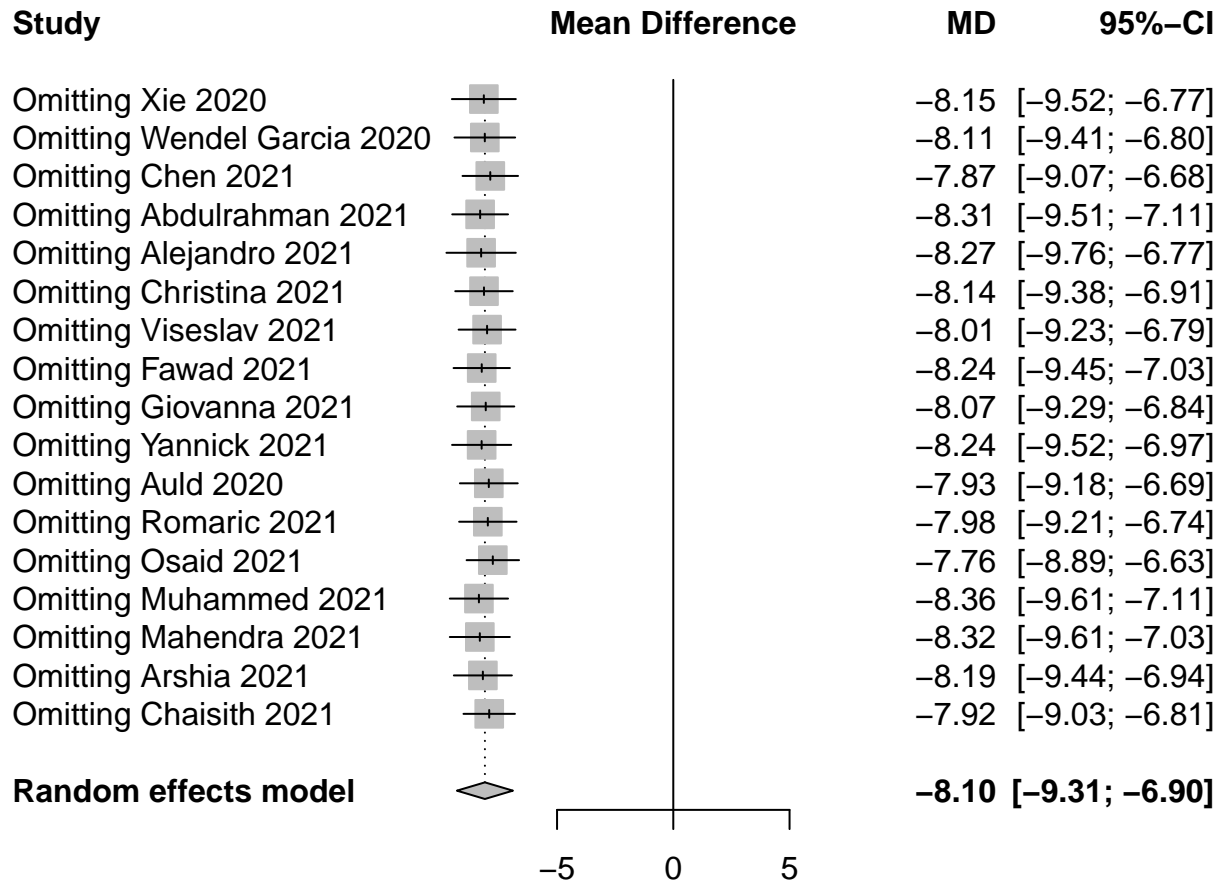

Supplement: Supplementary file 1 [file Data_Sheet_1.ZIP › Supplementary Material/Supplement 6.Sensitivity Analysis/age.pdf]

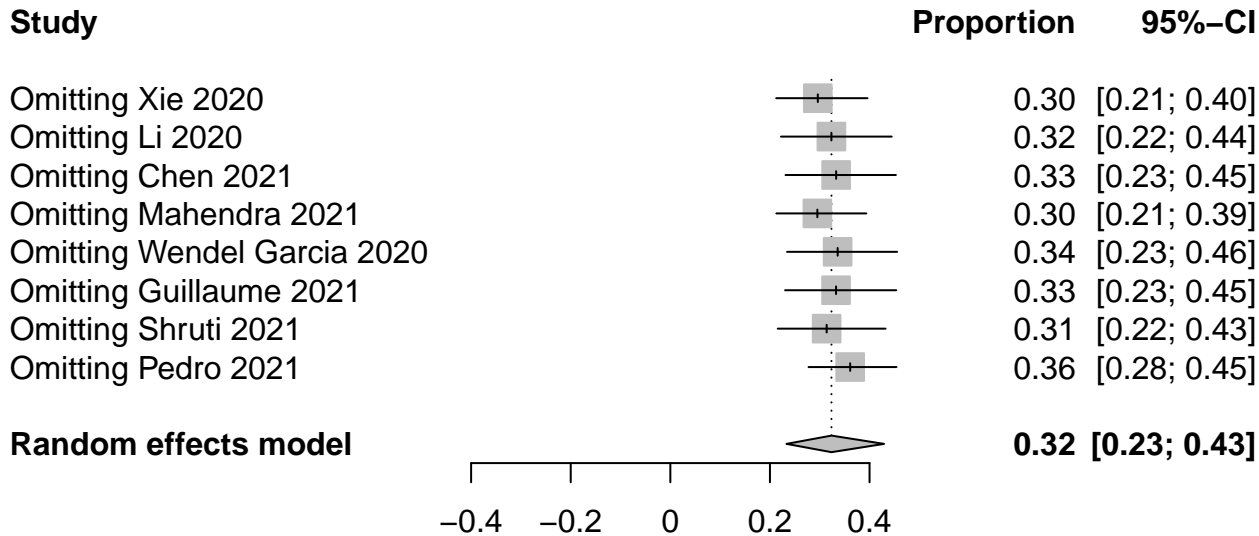

Supplement: Supplementary file 1 [file Data_Sheet_1.ZIP › Supplementary Material/Supplement 6.Sensitivity Analysis/hospital_motality-sen.pdf]

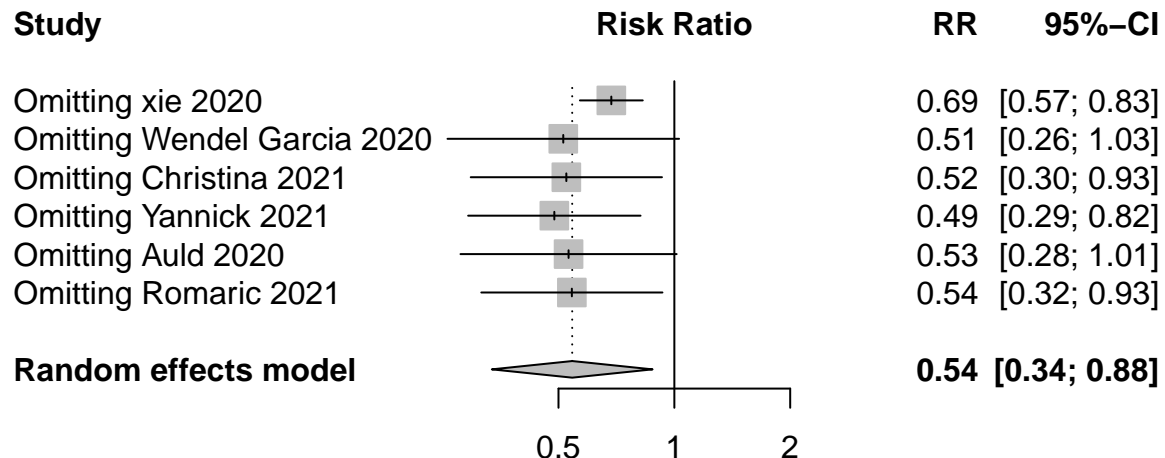

Supplement: Supplementary file 1 [file Data_Sheet_1.ZIP › Supplementary Material/Supplement 6.Sensitivity Analysis/vasoconstrictor.pdf]

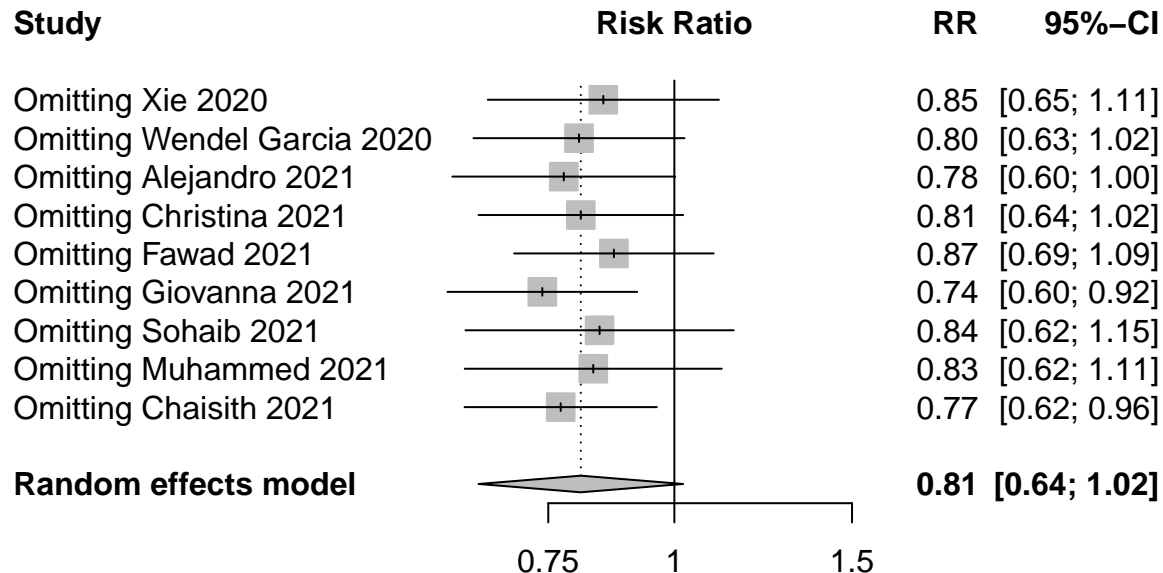

Supplement: Supplementary file 1 [file Data_Sheet_1.ZIP › Supplementary Material/Supplement 6.Sensitivity Analysis/ventilation.pdf]
